# Sandpile Universality in Social Inequality: Gini and Kolkata Measures

**DOI:** 10.3390/e25050735

**Published:** 2023-04-28

**Authors:** Suchismita Banerjee, Soumyajyoti Biswas, Bikas K. Chakrabarti, Asim Ghosh, Manipushpak Mitra

**Affiliations:** 1 Economic Research Unit, Indian Statistical Institute, Kolkata 700108, India; 2 Department of Physics, SRM University-AP, Amaravati 522240, India; 3 Condensed Matter Physics, Saha Institute of Nuclear Physics, Kolkata 700064, India; 4 Department of Physics, Raghunathpur College, Purulia 723133, India

**Keywords:** social inequality, gini index, kolkata index, sandpile model, self-organized criticality

## Abstract

Social inequalities are ubiquitous and evolve towards a universal limit. Herein, we extensively review the values of inequality measures, namely the Gini (*g*) index and the Kolkata (*k*) index, two standard measures of inequality used in the analysis of various social sectors through data analysis. The Kolkata index, denoted as *k*, indicates the proportion of the ‘wealth’ owned by (1−k) fraction of the ‘people’. Our findings suggest that both the Gini index and the Kolkata index tend to converge to similar values (around g=k≈0.87, starting from the point of perfect equality, where g=0 and k=0.5) as competition increases in different social institutions, such as markets, movies, elections, universities, prize winning, battle fields, sports (Olympics), etc., under conditions of unrestricted competition (no social welfare or support mechanism). In this review, we present the concept of a generalized form of Pareto’s 80/20 law (k=0.80), where the coincidence of inequality indices is observed. The observation of this coincidence is consistent with the precursor values of the *g* and *k* indices for the self-organized critical (SOC) state in self-tuned physical systems such as sand piles. These results provide quantitative support for the view that interacting socioeconomic systems can be understood within the framework of SOC, which has been hypothesized for many years. These findings suggest that the SOC model can be extended to capture the dynamics of complex socioeconomic systems and help us better understand their behavior.

## 1. Introduction

The distribution of wealth has been a topic of discussion and concern throughout human history. There is nothing more unequal than the distribution of wealth. No physical quantity can be perceived without sophisticated measuring instruments within the span wealth, which can vary by roughly nine orders of magnitude. This perception of wealth inequality has led to social discord since ancient times, to the extent that the history of popular struggles is broadly that against wealth inequality. In his dialogue (The Law) describing the ideal settlement, Magnesia, Plato recommended that the ratio of wealth between the poor and wealthy should exceed 1:4. However, societies have been far more unequal than that; therefore, herein, we would like to review the universal and extreme level of inequality in our society.

For the purpose of our discussion, we broaden our scope to include the study of inequalities in assets earned through competitive means, which can also include revenues earned through movies, citations of publications (of individuals, universities, journals, etc.), gains from stock market fluctuations, etc. As we shall see, in all such cases, inequalities are ubiquitous. However, the consequences are not similar. Wealth inequalities have a variety of severe social consequences, for example, limiting access to basic needs such as food, housing, healthcare, as well as access to education, thereby limiting opportunity for upward mobility, etc. On the other hand, inequality in, for example, movie revenues, is of very little consequence for the larger society. Therefore, while policies exist to moderate wealth inequalities in some cases, in other cases, the dynamics are completely unrestricted. Hence, such dynamics provide a window into what may occur with respect to inequalities in the distribution of some form of asset when such assets are sought after by competing entities without any restriction.

We argue, however, that irrespective of the particular context, dynamics of inequality follow broadly universal characteristics. It is, therefore, possible to shed light on a highly complex situation of wealth inequality by characterizing its universal dynamics. This characterization, of course, requires quantification of inequality through unambiguous and possibly intuitive measures. We attempt to do just that in this article. We study a novel inequality index; compare it with other existing inequality indices; and characterize the universal dynamics, followed by those indices, by analyzing real data and apply the same process to self-organized critical sand pile models driven by the production of entropy.

## 2. Social Inequality and Its Measures

Over a century ago (in 1896), Vilfredo Pareto [1] noticed that only 20% of Italy’s people possessed 80% of the country’s wealth. He proceeded to conduct surveys in other European nations, where he discovered, to his astonishment, that the same distribution held. The Pareto Principle, also called Pareto’s 80/20 law, asserts that 20% of the causes are responsible for 80% of the outcomes. In other words, the principle suggests that a small fraction of the factors contribute to a large majority of the results. The Pareto Principle has been used to analyze many different areas, from economics to quality management, even in personal development. In business, it is often used to identify the most important areas for improvement. For example, if a company wants to improve customer satisfaction, it can use the Pareto Principle to identify the 20% of customers who are responsible for 80% of the complaints.

**Figure 1:**
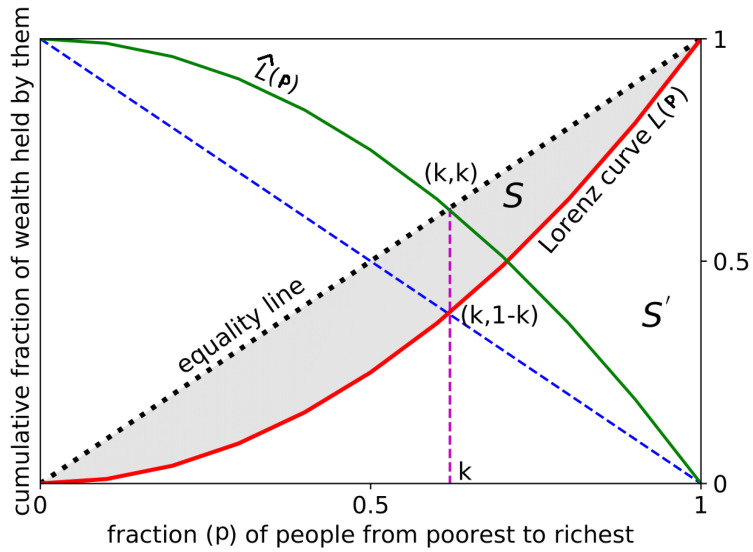
The Lorenz curve or function (L(p), red) shows the proportion of total wealth owned by a fraction (*p*) of people in ascending order of wealth. The black dotted line represents a scenario of perfect equality in which everyone possesses the same amount of wealth. The Gini index (*g*) is calculated as the area (*S*) between the Lorenz curve and the equality line (shaded region), normalized by the total area under the equality line ($S+S^{{}^{\prime }}=\frac{1}{2}$). The complementary Lorenz function ($\hat{L}\left(p\right)\equiv 1-L\left(p\right)$ is) shown in green. The Kolkata index (*k*) is determined by the point at which the Lorenz curve intersects the diagonal line perpendicular to the equality line. The value of $\hat{L}\left(k\right)=1-L\left(k\right)$ is equal to *k*, which indicates that *k* is a fixed point of $\hat{L}\left(p\right)$ and indicates the proportion of wealth owned by the top (1−k) fraction of the population.

In the year 1905, an American economist by the name of Max Lorenz [2,3] came up with the Lorenz curve, which is a graphical representation of the distribution of wealth in a society. If the population of a society is arranged in the ascending order of their wealth, then the curve is created by plotting the cumulative fraction of the wealth (L(p)) possessed by the *p* fraction of the poorest individuals (red curve shown in Figure 1).

If wealth were perfectly equally distributed, the Lorenz curve would be a straight line from the origin to the top right corner of the graph (black dotted line in Figure 1). In reality, the curve is usually downward-slanting, indicating that a relatively small portion of the population holds a disproportionate share of the wealth based on the fraction of people who possess wealth less than or equal to a certain amount, denoted as F(p), and the fraction of total wealth possessed by those people, denoted as L(p). To illustrate this, consider a society of *N* individuals with wealth distribution defined by the function f(y), where *y* represents the wealth of each person. The fraction of individuals with wealth less than or equal to *p* is calculated as the integral of f(y) from 0 to *p*, divided by *N* as follows:

(1) $$F\left(p\right)=\frac{1}{N}\int_{0}^{p}f\left(y\right)\;dy$$

The fraction of total wealth possessed by those individuals is calculated as the integral of yf(y) from 0 to *p*, divided by the total wealth of the society (μ) as follows:

(2) $$L\left(p\right)=\frac{1}{\mu }\int_{0}^{p}yf\left(y\right)\;dy.$$

Lorenz originally produced a graphical representation of L(p) plotted against F(p) using a parametric method. Both L(p) and F(p) are functions that increase monotonically and continuously from zero to one as *p* ranges from zero to infinity. The resulting plot, now known as the Lorenz curve, can be displayed within a unit square, which is depicted in a schematic form in Figure 1.

Mathematically, the Lorenz curve can be formulated in a more compact way as follows,

(3) $$L\left(p\right)=\frac{1}{\mu }\int_{0}^{p}F^{-1}\left(x\right)\;dx$$

where $F^{-1}\left(x\right)=\inf\left\{y|F\left(y\right)\geq x\right\}$, which means it will take the minimum value of *y* for which F(y)≥x.

Along with the definition of the Lorenz curve, for economic inequality, another kind of Lorenz curve is utilized, which is known as the ‘Complementary Lorenz curve’ ($\hat{L}\left(p\right)$; green curve in Figure 1). The complementary Lorenz curve is a plot that represents the distribution of wealth in a society in terms of the fraction of the total wealth held by the richest fraction of the population. In contrast to the standard Lorenz curve, which shows the fraction of the total population holding a given fraction of the total wealth, the complementary Lorenz curve shows the fraction of the total wealth held by the top fraction of the population. The complementary Lorenz curve is often used in the study of income and wealth inequality, as it provides a different perspective on the distribution of wealth in a society. For example, it can be used to compare the wealth held by the top 1% of the population to the wealth held by the bottom 99% of the population. In a society with a perfectly equal distribution of wealth, the standard Lorenz curve and the complementary Lorenz curve would be the same and would be represented by the 45-degree line. In a society with a high degree of inequality, the complementary Lorenz curve would be steeper than the 45-degree line, indicating that a large fraction of the total wealth is held by a small fraction of the population.

Therefore, to a certain extent, the Lorenz curve can be used to measure inequality. While the Lorenz curves contain the complete information about the wealth distribution and, consequently, that about wealth inequality, it is not often convenient to deal with the full distribution function, particularly when a comparative analysis or ranking is required. This calls for some form of a summary statistics to be drawn from the Lorenz curves. For this reason, in 1912, the Italian statistician and sociologist Corrado Gini developed the Gini index [4]. The Gini index is a widely used metric to measure statistical dispersion in the distribution of wealth or income among residents of a nation. It is commonly used to assess levels of inequality in a society. To compute the Gini index, the area between the Lorenz curve and the line of equality is divided by the total area under the line of equality. This ratio provides a numerical value that represents the degree of inequality present in the distribution of wealth or income. In Figure 1, the Gini index is

$$g=\frac{S}{\left(S+S^{{}^{\prime }}\right)}.$$

Equivalently, the Gini index is twice the area between the Lorenz curve and the line of perfect equality,

(4) $$g=2\int_{0}^{1}\left(p-L\left(p\right)\right)\;dp=1-2\int_{0}^{1}L\left(p\right)\;dp.$$

The Gini index ranges from 0 to 1, with 0 representing perfect equality and 1 representing perfect inequality. The Gini index is used by economists to measure the income inequality of a nation and is sometimes used to measure the inequality of other variables, such as wealth distribution and health inequality. The significance of adopting alternative measures of inequality is commonly recognized because no single summary statistic can capture all characteristics of inequality displayed by the Lorenz curve and the Gini index. In particular, the Gini index is known to be a rather non-intuitive measure, i.e., quoting its value does not provide an immediate picture of the underlying inequality. Indeed, the problem is deeper than intuition. Multiple Lorenz curves can have the same Gini index value. This means that calculating Gini index does not uniquely define inequality.

One major factor contributing to economic inequality is socialization or the tendency for the rich to become richer. This can occur because those with wealth have more resources to invest and accumulate more wealth, while those without wealth may struggle to get ahead. As a result, the gap between the rich and the poor can grow over time. The industrial revolution transformed economic interactions. Shops sold things produced more efficiently by factories. Economic socialization followed the industrial revolution. The automobile and telephone made travel and communication easier. Economic socialization changed during the Great Depression. As employment disappeared and wages diminished, people had to find other methods to make money. To help people cope with the economic crisis, unemployment insurance and welfare benefits were created. World War II reshaped economic socialization by the mid-20th century. The US standard of living rose as more commodities were available. Wealth spurred consumerism and the service industry. Socialization during this time gave more people the chance to rise economically and partake in the prosperity. The wealthy had more resources, jobs, and education than the poor. The global Gini (*g*) index value increased during this period, and in 1980–1990, this value remained at g≃0.65 (see [5]), i.e., the top 35% of the population earned almost 65% of the overall income. US tax policy favored the wealthiest, allowing them to pay lower rates than the middle and lower classes, increasing wealth disparity. Economic policies, such as taxation, subsidies, and other economic interventions, began to impact residents’ economic behavior at this time. In conclusion, economic inequality is a complex issue with many factors contributing to its persistence and growth. While tools such as the Lorenz curve and Gini index help to understand and quantify inequality, addressing the root causes of this issue will require a more comprehensive approach.

In recent years, there has been a growing concern about the increasing concentration of wealth in the hands of a small percentage of the population. In 2011, the ‘Occupy Wall Street’protest [6] was a response to this trend, with participants calling for greater economic equality and a fairer distribution of wealth. The movement consisted of a campaign of civil resistance, with protesters occupying public spaces in cities across the country to demand changes to the economic and political system. The movement began on 17 September 2011, when a group of protesters entered and began an occupation of Zuccotti Park in New York City. The protests quickly spread to other cities in the United States and around the world, inspiring similar protests in countries such as the United Kingdom, Spain, and Greece. The movement has been largely credited with helping to spark the global Occupy movement, which has seen occupations in many countries. The movement also served as a catalyst for a wide range of progressive issues, including income inequality, corporate greed, and the power of the financial sector.

The protesters argued that unless something were done to address economic inequality, 99% of the wealth would soon be possessed by just 1% of the population. The most famous slogan of the Occupy Wall Street movement was “We are the 99%”. This slogan was used to highlight the inequality that exists between the wealthiest 1% of the population and the other 99%. The slogan was meant to emphasize that the economic system is broken and that the wealthiest 1% are exploiting the resources of the other 99%. The slogan has since been adopted by various movements around the world and is still in use today.

Now, the question is: is the disparity really this great? In reality, is it possible to accurately determine what amount of the population possesses exactly what amount of wealth? To find the answers of these queries, in 2014, a new social inequality measure was introduced, name *k*-index or Kolkata index [7,8,9], providing the *k* fraction of wealth, citations, or vote shares possessed, attracted, or obtained by the richest or most successful (1−k) fraction of people, papers, or election candidates, respectively (see also [10] and references therein). In Figure 1, the k point is the *k* index. Mathematically, we can say that the *k* index for any income distribution is defined by the solution to the following equation:

(5) k+L(k)=1.

Extensive data analysis implies that a *k* value of more than the Pareto value (0.80) across competitive economies (where welfare measures are withdrawn), citation shares in top-ranked universities or among successful individual scientists, or vote shares in vibrant democratic elections has perhaps often continued for ages beyond our notice. Indeed, observations suggest that the inequality measure (*k*) can be as high as 0.86, which is more than the Pareto value (*k* = 0.80) but less than many apprehended limits or conjectures, such as that in the ‘Occupy Wall Street’ slogan (*k* = 0.99). In short, data analysis suggests that almost 14% of people, papers, election candidates, or even wars possess, attract, capture, or cause about 86% of the wealth, citations, votes, or deaths, respectively. For example study shows that about 12% of the 2386 publications (books, documents, letters, etc.) by Karl Marx, as collected in his Google Scholar page maintained by the British National Library, accounts for about 88% of the total 424,810 citations collected on that page as of today.

Furthermore, in 2016, Watkins et. al. wrote a review paper [11] on the evolution of social systems to a self-organized critical state. Social systems evolve in complex and unpredictable ways. However, many theorists have suggested that social systems tend to move towards a state of self-organizing criticality, or an SOC state (see [12,13]). This is a state in which the system is balanced between order and chaos, allowing for a dynamic and adaptive response to changing environmental and social conditions. Such self-organization can result in emergent properties, such as the emergence of group norms, the development of new practices or technologies, and the emergence of social networks that can facilitate collective action. Ultimately, the exact form that a social system takes is dependent upon a variety of factors, including the available resources and the environmental and social conditions. Examples of an SOC include earthquakes, forest fires, and the stock market.

Therefore, in his book published in 2017 [14], Piketty highlights the fact that the wealth of the top 10% of the population is continuously expanding at an alarming rate. He argues that there is a tendency for the rate of return on capital to be higher than the rate of economic growth, which leads to the concentration of wealth among a small number of individuals. He suggests that this concentration of wealth poses a threat to democratic societies, as it can lead to social and political instability.

In 2020, extensive data analysis [15] showed that as competition increases in various social institutions, including markets, universities, and elections, the values of the two general inequality indices (the Gini (*g*) and Kolkata (*k*) indices), approach one another. It was further demonstrated that under conditions of unrestricted competition, these two indices equalize and stabilize at a certain value ($k_{max}≃0.87≃g_{max}$). We suggest interpretation of this coincidence of inequality indices as a broader application of Pareto’s 80/20 rule (k=0.80). Additionally, the synchronicity of the inequality indices noted here is strikingly comparable to that of self-organized critical (SOC) systems previously discussed. The findings outlined the following sections provide quantitative evidence for the long-held hypothesis that interacting socioeconomic systems can be observed within the context of SOC systems.

Few social inequality indicators have been explored in relation to the subcritical dynamical properties (measured in terms of the avalanche size distributions) of some SOC models as their respective stationary critical states approach. A recent study [16] (2022) showed that different models of self-organized criticality (SOC), including the Bak–Tang–Wiesenfeld (BTW) sand pile, the Manna sand pile, the quenched Edwards–Wilkinson interface (EW) model, and the fiber bundle interface (FBM), all display a similar precursor to SOC state values of inequality measures, such as the Gini and Kolkata indices. These results suggest that SOC systems share a high degree of commonality when it comes to indicators of inequality. Additionally, comparing these findings to similar results from socioeconomic systems with unrestricted competitions, it appears that inequality may emerge due to proximity to SOC states. Specifically, the *k* index for SOC models appears to be around ≃0.86. These observations provide further evidence for the universality of inequality measures across various physical and socioeconomic systems. We parallelly numerically demonstrated that the cluster or avalanche size distributions in the various SOC models of self-tuned physical systems (also argued to model all social systems; see, e.g., [11]) do reach a similar *k* value as the respective SOC points are approached, indicating that as one approaches the SOC point, about 86% of the avalanche mass is carried away by 14% of the avalanches. This is similar to the inequalities that we observed both mathematically and empirically for different socioeconomic systems.

**Figure 2:**
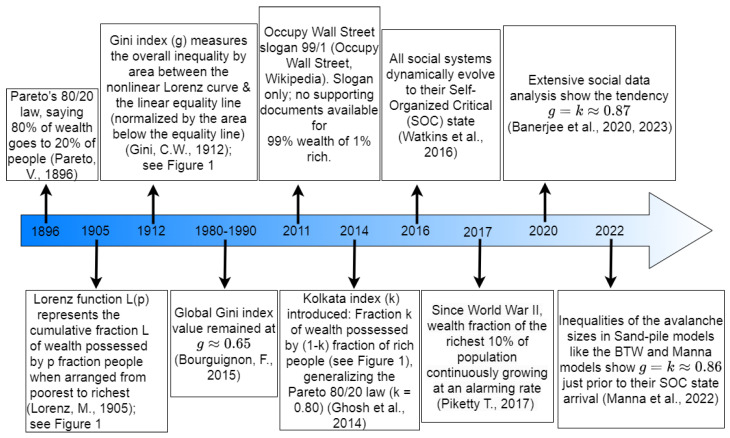
Timeline of the evolution of social inequality measures since 1896 and their universal convergence to those for sand pile models prior to their respective self-organized critical (SOC) points. We start the timeline from 1896 with the work of Pareto [1] and subsequent developments in 1905 by Lorenz [2] and Gini [4] in 1912. Then we observe the consistency of the Gini index (*g*) for a decade-span 1980–1990 [5]. Subsequent protest happened in 2011 at the Wall Street for the advection of the majority portion of the entire wealth in the hands of very few people [6]. In 2014, Kolkata index (*k*) was introduced as another measure of inequality in the wealth distribution [7]. In 2016, Watkins and others proposed that all social systems evolve towards the respective SOC state [11]. Piketty (2017) pointed out forcefully about the continuous growth of the wealth of top 10% of the people [14]. In the year 2020, the work of Banerjee and others reported that the inequality of the social systems has a tendency to evolve at a point of g=k≈0.87 [9,15]. In 2022, Manna and others showed numerically that many physical SOC systems show g=k≈0.86 just preceding the SOC points in the respective systems [16]. In this review, the figures and tables are arranged with self-contained captions in an attempt to provide readers with an overview of our motivation and the main results presented the introductory and concluding sections (15 figures and 13 tables and their captions).

Figure 2 shows a schematic representation of this timeline since 1896 at a glance, as discussed above.

## 3. Calculating the Inequality Indices

In the last section, we defined the Lorenz function and the two inequality measures that summarize the inequality statistics of a society (Gini and Kolkata indices). In this section, we proceed to calculate these quantities and attempt to find analytical relations between them.

### 3.1. Properties of the Lorenz Curve

With the definition of Lorenz curve, as mentioned before, we can enumerate several of the properties that such a curve must follow:

1. The Lorenz curve range from {F(p),L(p)}≡{0,0} to {1,1}.
  **Proof.**  Equation (3) shows see that at p=0, F(0)=0 and L(0)=0. Similarly, for p=1, we have F(1)=1 and L(1)=1. Hence, as p∈[0,1], the Lorenz curve always ranges from {F(p),L(p)}≡{0,0} to {1,1}. □
2. The Lorenz curve is a concave and monotonically increasing function of wealth.
  **Proof.**  (6) $$\frac{d\;L(p)}{d\;p}=\frac{f\;p}{\mu }$$
  and,
  (7) $$\frac{d\;F(p)}{d\;p}=\frac{f}{N},$$
  so the slope of the Lorenz curve is given by,
  (8) $$\frac{d\;L(p)}{d\;F(p)}=\frac{N}{\mu }\;p$$
  As p∈(0,1) and is always increasing, Equation (6) shows that the slope of the Lorenz curve is always increasing. Hence, the Lorenz curve is a concave and monotonically increasing function of wealth. □
3. The Lorenz curve for a society in which each person possesses an equal amount of wealth is a diagonally sloping line.
  **Proof.**  If each person in a society possesses an equal amount of wealth, wealth distribution follows a Dirac delta function as,
  $$f\left(y\right)=\delta \left(y-y_{0}\right)$$
  Now,
  $$\begin{array}{cc} F(p) & =\frac{1}{N}\int_{0}^{p}f\left(y\right)\;dy\\ \; & =\frac{1}{N}\int_{0}^{p}\delta \left(y-y_{0}\right)\;dy. \end{array}$$
  Therefore,
  $$F\left(p\right)=\left\{\begin{array}{c} 0\;\mathrm{if}\;p<y_{0}\\ 1\;\mathrm{if}\;p\geq y_{0} \end{array}\right.$$
  Again,
  $$L\left(p\right)=\left\{\begin{array}{c} 0\;\mathrm{if}\;p<y_{0}\\ 1\;\mathrm{if}\;p\geq y_{0} \end{array}\right.$$
  Hence, we can see that Lp=F(p) i.e., the Lorenz curve is a diagonal line for this particular case. □
4. The upper limit of the Lorenz curve is bounded by the equality line.
  **Proof.**  According to the second derivative of Equation (6),
  $$\frac{d^{2}L}{dF^{2}}=\frac{N}{\mu }{\left(\frac{d\;F}{d\;y}\right)}^{-1}=\frac{N}{\mu }\frac{N}{f}\geq 0$$
  Therefore, one can conclude from the above exercise that the Lorentz curve can never exceed the diagonal line. Moreover, L=F=0 when p=0 and L=F=1, as p→∞. In addition, the concave topology of the Lorentz curve indicates that it is bounded by the diagonal line (also known as the egalitarian line). □

### 3.2. Exemplary Calculations of the Lorenz Curve

Here, we show the calculation of the Lorenz curve for some simple wealth distribution functions, albeit continuous and discrete.

1. *Uniform wealth distribution:* Let us examine a society in which the distribution of income is uniform over a finite range of values within the interval [a,b], where 0<a<b<1. The corresponding probability density function is given by $f_{u}\left(x\right)=\frac{1}{\left(b-a\right)}$, and the cumulative distribution function is $F_{u}\left(x\right)=\left(x-a\right)/\left(b-a\right)$ for all values of *x* within [a,b]. Applying Equation (3), we obtain the following Lorenz curve for this distribution:
  (9) $$L_{u}\left(p\right)=\frac{1}{\mu_{u}}\int_{0}^{p}\left\{a+\left(b-a\right)q\right\}\;dq=p\left[1-\frac{\left(b-a\right)}{\left(a+b\right)}\left(1-p\right)\right],$$
  The distribution has a mean of $\mu_{u}=\frac{\left(a+b\right)}{2}$, and $F_{u}^{-1}\left(q\right)=a+\left(b-a\right)q$. It is worth noting that if a=0, the Lorenz curve simplifies to $L_{\bar{u}}\left(p\right)=p^{2}$.
2. *Exponential wealth distribution:* Let us consider an exponential income distribution characterized by the probability density function $f_{E}\left(x\right)=\lambda e^{-\lambda x}$, where λ>0, and the cumulative distribution function $F_{E}\left(x\right)=1-e^{-\lambda x}$ for all x≥0. The mean of this distribution is given by $\mu_{E}=\frac{1}{\lambda }$, and $F_{E}^{-1}\left(q\right)=-\left(\frac{1}{\lambda }\right)\ln\left(1-q\right)$. The Lorenz curve for this distribution is therefore given by:
  (10) $$L_{E}\left(p\right)=\int_{0}^{p}-\ln\left(1-q\right)\;dq=p-\left(1-p\right)\ln\left(\frac{1}{1-p}\right).$$
3. *Pareto wealth distribution:* Let us now consider a society with a Pareto-like income distribution. The probability density function for this distribution is given by $f_{P,\alpha }\left(x\right)=\frac{\alpha {\left(m\right)}^{\alpha }}{{\left(x\right)}^{\alpha +1}}$, and the cumulative distribution function is $F_{P,\alpha }\left(x\right)=1-{\left(\frac{m}{x}\right)}^{\alpha }$, where m>0 is the minimum income, α>0, and the probability density and cumulative distribution functions are defined for all x≥m. The mean of this distribution is $\mu_{P}=\frac{\alpha m}{\left(\alpha -1\right)}$, and $F_{P,\alpha }^{-1}\left(q\right)=m{\left(1-q\right)}^{-(\frac{1}{\alpha })}$, which gives the Lorenz curve as follows:
  (11) $$L_{P,\alpha }\left(p\right)=\frac{\left(\alpha -1\right)}{\alpha }\int_{0}^{p}{\left(1-q\right)}^{-(\frac{1}{\alpha })}\;dq=1-{\left(1-p\right)}^{1-\frac{1}{\alpha }}.$$
4. *Discrete wealth distribution:* To obtain the Lorenz function for a discrete income distribution, consider an economy comprising G groups of people, where each group (g) comprises $n_{g}$ individuals with the same income ($x_{g}$) such that $0\leq x_{1}\leq x_{2}\leq \cdots \leq x_{G}$. The total population of the economy is N, and the total income is M, leading to a mean income of $\mu_{g}=M/N$. The income distribution is a discrete random variable (X) with a probability mass function of $f_{G}\left(x_{g}\right)=n_{g}/N$ for all g ranging from 1 to G and a distribution function ($F_{G}\left(x\right)$) defined by 0 if $x\in [0,x_{1})$, $f_{G}\left(x\right)=n_{g}/N$ if $x\in [x_{g},x_{g+1})$ for any given g ranging from 1 to (G−1), and 1 if $x\geq x_{G}$. We define N(g) and M(g) as the cumulative proportion of the population and cumulative proportion of the total income, respectively, for each group (g). For any given g ranging from 1 to G and any $q_{g}\in \left(N\left(g-1\right),N\left(g\right)\right]$, it can be verified that $F_{G}^{-1}\left(q_{g}\right)=x_{g}$. Using the Lorenz function formula, we can calculate the Lorenz function ($L_{F_{G}}\left(p_{g}\right)$) for any given g ranging from 1 to *G* and any $p_{g}\in \left(N\left(g-1\right),N\left(g\right)\right]$ as,
  (12) $$L_{F_{G}}\left(p_{g}\right)=M\left(g-1\right)+\left(p_{g}-N\left(g-1\right)\right)\frac{Nx_{g}}{M}.$$
  We make two observations in this context. The first observation states that the Lorenz function is piecewise linear, which means that it is composed of several line segments. The kink points represent the points where the direction of the Lorenz curve changes; they occur at the boundaries of each income group. At these points, there is a jump in the cumulative share of income that is distributed to each group, which causes a change in the slope of the Lorenz curve. The second observation is that if there is only one income group in the economy, then the Lorenz curve is a straight line passing through the origin, with a slope of 1. This means that the distribution of income is perfectly equal, and each individual in the economy has the same income. In this case, the Lorenz curve coincides with the diagonal of the unit square, which represents the line L(p)=p.

### 3.3. Properties of the Gini Index

The above definition also implies several properties that are important to understand when interpreting and using this index. Some of the key properties of the Gini index are:

1. *Range:* The Gini index ranges from 0 to 1, with 0 indicating complete equality (i.e., everyone has the same income or wealth) and 1 indicating complete inequality (i.e., one person has all the income or wealth);
2. *Normalization:* The Gini index is normalized, meaning that it can be used to compare inequality across different populations or over time. This allows for meaningful comparisons even when the populations or time periods have different sizes or levels of income;
3. *Sensitivity to changes in the distribution:* The Gini index is sensitive to changes in the distribution of income or wealth, meaning that even small changes in the distribution can result in large changes in the Gini index. This property makes the Gini index a useful tool for measuring the impact of policies or events that affect the distribution of income or wealth;
4. *Unimodality:* The Gini index is unimodal, meaning that it has a single peak. This property allows for the ranking of populations or time periods based on their level of inequality;
5. *Invariance to scale:* The Gini index is invariant to scale, meaning that it is not affected by changes in the units of measurement (e.g., dollars, euros, etc.). This allows for meaningful comparisons of inequality across populations or time periods using different currencies.

### 3.4. Exemplary Calculations of the Gini Index

Here, we show calculations of the Gini index for some simple continuous and discrete wealth distributions.

1. *Uniform wealth distribution:* For a uniform distribution on a compact interval [a,b], following 0≤a<b<∞ leads to the following Gini index,
  (13) $$g_{u}=2\int_{0}^{1}\left[q-q\left\{1-\frac{\left(b-a\right)}{\left(a+b\right)}\left(1-q\right)\right\}\right]\;dq=\frac{\left(b-a\right)}{3(a+b)}.$$
2. *Exponential wealth distribution:* An exponential distribution of the form $F_{E}\left(x\right)=1-e^{-\lambda x}$ for any x≥0 and λ>0 leads to the following Gini index,
  (14) $$g_{E}=2\int_{0}^{1}\left[q-L(q)\right]\;dq=2\int_{0}^{1}\left(1-q\right)\ln\left(\frac{1}{1-q}\right)\;dq=\frac{1}{2}.$$
3. *Pareto wealth distribution:* A Pareto distribution of the form $F_{P,\alpha }\left(x\right)=1-{\left(m/x\right)}^{\alpha }$ with m>0 as the minimum income and α>1 results in a Gini index of the following form,
  (15) $$g_{P,\alpha }=2\int_{0}^{1}\left[q-\left\{1-{\left(1-q\right)}^{1-\frac{1}{\alpha }}\right\}\right]\;dq=\frac{1}{2\alpha -1}.$$
  As we graph the Gini index for various values of α, where α is greater than 1, we observe that as α increases, the Gini index decreases. Additionally, as α approaches 1, the Gini index tends towards 1. Furthermore, if we set $\hat{\alpha }$ to be equal to $\frac{\ln5}{\ln4}$, then the Gini index for $g_{P,\alpha }$ is approximately 0.7565.
4. *Discrete wealth distribution:* Consider the discrete random variable $F_{G}$ discussed previously for which the Lorenz function is given by Equation (12). Accordingly, we have the following explicit form of the Gini index,
  (16) $$g_{F_{G}}=\frac{\sum_{g=1}^{G}\sum_{t=1}^{G}n_{t}n_{g}\left|x_{t}-x_{g}\right|}{2\;N\;M}.$$
  Note that if $n_{g}=1$ for all g∈{1,…,G} so that G=N and $M=\sum_{g=1}^{N}x_{g}$, then it follows from Equation (16) that,
  (17) $$g_{F_{G}}=\frac{\sum_{g=1}^{G}\sum_{t=1}^{G}\left|x_{t}-x_{g}\right|}{2\;N\;\sum_{g=1}^{N}x_{g}}.$$

### 3.5. Properties of the k-Index

With the above definition, the *k* index has several characteristics, which are listed below:

1. The *k* index is a unique fixed point of the complementary Lorenz function.
  **Proof.**  We can rewrite Equation (5) as,
  (18) $$k=1-L\left(k\right)=\hat{L}\left(k\right).$$
  Hence, the *k* index is a fixed point of the complementary Lorenz function. Since the complementary Lorenz function maps [0, 1] to [0, 1] and is continuous (as shown in Figure 1), it has a fixed point according to Brouwer’s fixed-point theorem. Furthermore, since $\hat{L}\left(p\right)$ is non-increasing, the fixed point has to be unique. □
2. For any distribution, k∈[1/2,1], and the normalized *k* index (K=1−2k) lies in the interval [0,1].
  **Proof.**  Observe that if L(p)=p, then, according to Equation (18), k=1/2, and for any other income distribution, 1/2<k<1. Also note that while the Lorenz curve typically has only two trivial fixed points, that is, L(0)=0 and L(1)=1, the complementary Lorenz function ($\hat{L}\left(p\right)$) has a unique non-trivial fixed point (*k*). Now, the normalized *k* index is given by K=1−2k, so if k∈[1/2,1], then K∈[0,1]. □
3. The *k* index as a generalization of the Pareto Principle.
  **Proof.**  The *k* index can be thought of as a generalization of the Pareto’s 80/20 rule. Note that L(k)=1−k; hence, the top 100(1−k)% of the population has 100(1−(1−k))=100k% of the income. Hence, the ‘Pareto ratio’ for the k index is k/(1−k). Note that this proportion is derived internally from the distribution of income, and typically, there is no expectation that it will align with the Pareto Principle. □
4. Interpreting the *k* index in terms of rich–poor disparity.
  **Proof.**  Let us split society into two groups: the ‘poorest’ group, consisting of a fraction (p) of the population, and the ‘rich’ group, consisting of a fraction (1−p) of the population. Using the Lorenz curve (L(*p*)), we can determine the distance between the “boundary person” and the poorest person on one hand and the distance between the “boundary person” and the richest person on the other hand. These distances can be calculated using the following equations: $\sqrt{p^{2}+L{\left(p\right)}^{2}}$ and $\sqrt{{\left(1-p\right)}^{2}+{\left(1-L\left(p\right)\right)}^{2}}$, respectively. The k index is a way of dividing society into two groups such that the boundary person is equidistant from the poorest and richest persons. The disparity function value at the k index is given by D(k)=k−1/2. This function measures the gap between the proportion (k) of the poor from the 50/50 population split. If society is not completely equal, then k>1/2, making it a useful tool to highlight the rich–poor disparity. In this case, k defines the income proportion of the top (1−k) proportion of the rich population. □
5. The *k* index as a solution to optimization problems.
  **Proof.**  The *k* index is the unique solution to the following surplus maximization problem:
  (19) $$k=argmax_{P\in [0,1]}\int_{0}^{P}\left(\hat{L}\left(t\right)-t\right)\;dt.$$
  The value of *k* is such that it maximizes the area between the complementary Lorenz function and the income distribution line linked with an egalitarian distribution for the lower-income population. Equation (19) is a consequence of the fact that $\hat{L}\left(p\right)\geq p$ for all p∈[0,k] and $\hat{L}\left(p\right)\leq p$ for all p∈(k,1]. Similarly, the *k* index is the only solution to the surplus minimization problem (which is the dual of the problem in (19)):
  (20) $$k=argmin_{P\in [0,1]}\int_{P}^{1}\left\{(1-t)-L(t)\right\}\;dt.$$
  Therefore, (1−k) is the fraction of the higher-income population for which the area between the income distribution line associated with the egalitarian distribution and the Lorenz function is minimized. □
6. To reduce inequality between groups, the *k* index is a better indicator.
  **Proof.**  The ordering of Lorenz curves based on the *k* index is not the same as the ordering based on the Gini index. While the Gini index is influenced by transfers only within the poor or rich population, the ranking based on the *k* index is influenced only by transfers between the two groups. This implies that if the objective is to reduce inequality between the groups, then the *k*-index is a more appropriate measure to use. □

### 3.6. Exemplary Calculations of the *k* Index

Here, we show calculations of some simple continuous and discrete wealth distribution functions. The majority of this subsection is adopted from [9].

1. *Uniform wealth distribution:* Consider a case in which the uniform distribution (*F*) is defined for [a,b], where 0≤a<b<∞. Then, the *k* index is given by,
  (21) $$k_{u}=\frac{\left(3a+b\right)+\sqrt{5a^{2}+6ab+5b^{2}}}{2(b-a)},$$
  and the normalized *k* index is given by,
  (22) $$K_{u}=\frac{-2\left(a+b\right)+\sqrt{5a^{2}+6ab+5b^{2}}}{\left(b-a\right)}.$$
2. *Exponential wealth distribution:* For the exponential distribution ($F_{E}$), the complementary Lorenz function is given by ${\hat{L}}_{E}\left(p\right)=\left(1-p\right)\left[1+\ln\left(\frac{1}{1-p}\right)\right]$. One can show that the *k*-index is $k_{E}≃0.6822$; hence, the normalized *k* index is $K_{E}≃0.3644$.
3. *Pareto wealth distribution*: For the Pareto distribution ($F_{P,\alpha }$), the complementary Lorenz function is given by $L_{P,\alpha }\left(p\right)={\left(1-p\right)}^{1-\frac{1}{\alpha }}$. The *k* index is therefore a solution to the following equation,
  (23) $${\left(1-k_{P,\alpha }\right)}^{1-\frac{1}{\alpha }}=k_{P,\alpha }.$$
  It is difficult to provide a general solution to this equation. However, we have an interesting observation in this context. If $\alpha =\frac{\ln5}{\ln4}≃1.16$, then the *k* index is $k_{P,\alpha }=0.8$, corresponding to the Pareto principle or the 80/20 rule. Also note that the normalized *k* index is $K_{P,\alpha }=0.6$.
4. *Discrete wealth distribution:* Consider any discrete random variable with the distribution function ($F_{G}$) discussed above for which the Lorenz function is given by Equation (12). To obtain the explicit form of the *k* index, one can first apply a simple algorithm to identify the interval of the form [N(g−1),N(g)) defined for g∈{1,…,G} in which the *k* index can lie.
  Since N(G)=M(G)=1, if we have N(G−1)+M(G−1)<1 in some step, then in the next step, this algorithm has to end, since N(G)+M(G)=2>1.
  Suppose that for any discrete random variable with the distribution function ($F_{G}$) discussed earlier, Algorithm-1 identifies $g^{*}\in \left\{1,\ldots ,G\right\}$ such that $N\left(g^{*}\right)+M\left(g^{*}\right)\geq 1$. If $N\left(g^{*}\right)+M\left(g^{*}\right)=1$, then $k_{F_{G}}=N\left(g^{*}\right)$, and if $N\left(g^{*}\right)+M\left(g^{*}\right)>1$, $k_{F_{G}}$ is the solution to the following equation:
  $$k_{F_{G}}+\left\{M\left(g^{*}-1\right)+\left(k_{F_{G}}-N\left(g^{*}-1\right)\right)\left(\frac{Nx_{g^{*}}}{M}\right)\right\}=1.$$
  Thus, to derive the *k* index of any discrete random variable with distribution function $F_{G}$, we first my identify the group $g^{*}\in \left\{1,\ldots ,G\right\}$ such that $k_{F_{G}}\in \left(N\left(g^{*}-1\right),N\left(g^{*}\right)\right]$ (using Algorithm 1); then, using $g^{*}$, we obtain the following value of $k_{F_{G}}$:
  (24) $$k_{F_{G}}=\left\{\begin{array}{cc} N(g^{*}) & \mathrm{if}\;N\left(g^{*}\right)+M\left(g^{*}\right)=1,\\ \frac{\mu_{G}+N\left(g^{*}\right)x_{g^{*}}-M\left(g^{*}\right)}{\mu_{G}+x_{g^{*}}} & \mathrm{if}\;N\left(g^{*}\right)+M\left(g^{*}\right)>1. \end{array}\right.$$

**Algorithm 1:**
Step 1:Consider the smallest $g_{1}\in \left\{1,\ldots ,G\right\}$ such that $N(g_{1})\geq 1/2$ and consider the sum of $N\left(g_{1}\right)+M\left(g_{1}\right)$. If $N\left(g_{1}\right)+M\left(g_{1}\right)\geq 1$, then stop, and $k_{F_{G}}\in \left(N_{g_{1}-1},N\left(g_{1}\right)\right]$; in particular, $k_{F}=N\left(g_{1}\right)$ if and only if $N\left(g_{1}\right)+M\left(g_{1}\right)=1$. Instead, if $N\left(g_{1}\right)+M\left(g_{1}\right)<1$, then go to Step 2, consider the group $g_{1}+1$, and repeat the process.

⋮
Step *t*:Having reached Step *t* means that in Step (t−1), we had $N\left(g_{1}+t-1\right)+M\left(g_{1}+t-1\right)<1$. Therefore, consider the sum of $N\left(g_{1}+t\right)+M\left(g_{1}+t\right)$. If $N\left(g_{1}+t\right)+M\left(g_{1}+t\right)\geq 1$, then, stop; $k_{F_{G}}\in \left[N\left(g_{1}+t-1\right),N\left(g_{1}+t\right)\right)$, and, in particular, $k_{F}=N\left(g_{1}+t\right)$ if and only if $N\left(g_{1}+t\right)+M\left(g_{1}+t\right)=1$. If $N\left(g_{1}+t\right)+M\left(g_{1}+t\right)<1$, then proceed to Step (t+1).

## 4. Analytical Studies on the Emerging Coincidence of the Gini and Kolkata Indices

### 4.1. A Landau-like Phenomenological Expansion of the Lorenz Function

Before going into specific forms of the Lorenz curve and their corresponding inequality indices, we will first outline an attempt to achieve a Landau-like phenomenological expansion of the Lorenz function that obeys the abovementioned properties (see, e.g., [17]). In particular, as a minimal non-trivial expansion, we could write

(25) $$L\left(p\right)=Ap+Bp^{2},$$

where A>0, B>0, and A+B=1. It then follows that the calculation of $g=1-2\int_{0}^{1}L\left(p\right)dp$ and k=1−L(k) gives

(26) $$k=\frac{\left(3g-2\right)\pm \sqrt{{\left(2-3g\right)}^{2}+12g}}{6g},$$

which, in the limit of g→0, gives k=1/2+3g/8, which implies that if a situation arises in which k=g, then k=g=0.8, which is the Pareto value. In later sections, we will discuss the actual situations and data in models under conditions of unrestricted competition.

### 4.2. Some Typical Power Law Forms of Lorenz Functions

An interesting observation is made when the Lorenz curve is parameterized through a power law curve in *p* and when the respective Lorenz curve’s Gini index and *k* index are plotted. When the power law index is increased, we observe that the values of the Gini and *k* indices converge towards each other and meet at a point around 0.87, which is not equal to 1.

Here, we consider a set of Lorenz functions with the functional form $L\left(p\right)=p^{n}$, where *p* ranges within the interval [0,1], and *n* is a positive integer greater than or equal to one. For this family of functions, we can derive the corresponding Gini index $g_{n}$, which is equal to (n−1)/(n+1). To investigate this relationship further, we tabulated the Lorenz functions (L(p)) for *n* values ranging from 1 to 20 and computed their associated Gini index ($g_{n}$) and *k* index ($k_{n}$). The results of our calculations are presented in Table 1.

**Figure 3:**
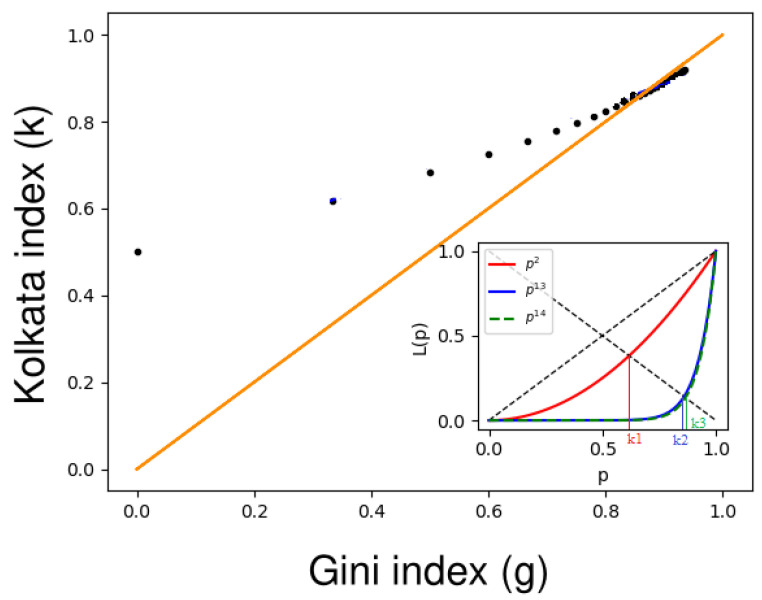
Graph of the Gini index (*g*) versus the *k* index (*k*), where the orange line represents the equality line (g=k). The black dots indicate the (g,k) values for the Lorenz function, $L\left(p\right)=p^{n}$, and *n* ranges from 1 to 20. The inset shows the Lorenz curves for $L\left(p\right)=p^{2}$ (red curve), $L\left(p\right)=p^{13}$ (blue curve), and $L\left(p\right)=p^{14}$ (green dashed curve), with their corresponding *k*-index values (k1≃0.618, k2≃0.860, and k3≃0.866, respectively). This figure is adopted from Banerjee et al. (2022) [15].

Table 1 shows that if n=13, then $g_{13}≃0.857<k_{13}≃0.860$, and if n=14, then $g_{14}≃0.867>k_{14}≃0.866$, implying that coincidence takes place for n∈(13,14) (see in Figure 3). The coincidence between the Gini index and the *k* index occurs at $n^{*}\in \left(n_{1}=13.82986,n_{2}=13.82987\right)$; hence,

(27) $$g_{n^{*}}=k_{n^{*}}\in \left(g_{n_{1}}=0.8651369,g_{n_{2}}=0.8651370\right)\in \left(\frac{6}{7},\frac{13}{15}\right).$$

The above study shows that there is no positive integer (*n*) for which the *g* and *k* index values coincide, and if *n* represents a positive real number, then there exists a value of $n=n^{*}$ for which these index values coincide.

### 4.3. Some Generic Forms of Lorenz Functions

**Figure 4:**
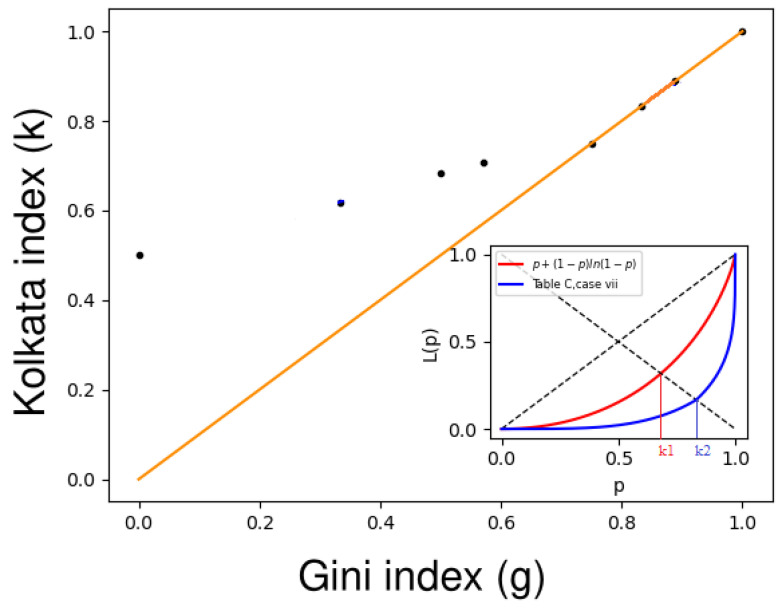
The plot of the Gini index (*g*) versus the *k* index (*k*), where the orange line corresponds to the g=k line. The black dots represent (g,k) values for several simple Lorenz functions, as listed in Table 2. The black dots tend to converge towards the g=k line higher values of *g*. In the inset, two different Lorenz curves are shown for cases (4) and (7) from Table 2. The red curve represents the Lorenz curve for case (4) with a *k*-index value of k1≃0.682, while the blue curve represents the Lorenz curve for case (7) with a *k*-index value of k2≃0.833. These results provide insight into the relationship between the Gini index and the *k* index, as well as the behavior of these measures across different Lorenz curves (adopted from [15]).

Therefore, with the above exercise, we observe a specific parameterized Lorenz function for which the Gini and *k* indices become equal. For more generic Lorenz functions, this equality of the Gini and *k* indices is exemplified in Table 2 (also see Figure 4).

In this review article, we examine the properties of finite polynomial Lorenz functions through the seven cases presented in Table 2. The first case (1) has already been addressed, where $a_{1}=\ldots =a_{n-1}=0$ and $a_{n}=1$. In case (2), $a_{1}=\ldots =a_{n}=1/n$, which results in a coincidence value of approximately 0.869 for some value between 65 and 66. This coincidence value is higher than that obtained in case (1). In case (3), $a_{1}=\left(n-1\right)/n$ and $a_{2}=\ldots =a_{n}=1/\left[n\left(n-1\right)\right]$. Since the weight $a_{1}$ is relatively high, *k* is close to 1/2; therefore, there is no coincidence between the Gini index and the *k* index. In case (4), $a_{n}=\left(n-1\right)/n$ and $a_{1}=\ldots =a_{n-1}=1/\left[n\left(n-1\right)\right]$, resulting in a coincidence value of approximately 0.874 for some value between 17 and 18, which is higher than that in case (2). In case (5), $a_{m}=\left[6m\left(n+1-m\right)\right]/\left[n\left(n+1\right)\left(n+2\right)\right]$ for all m=1,2,…,n, and the coincidence value is 0.874 for some number between 40 and 41, which is not an improvement compared to case (4). In case (6), $a_{m}=\left[2\left(m+1\right)\right]/\left[n\left(n+3\right)\right]$ for all m=1,2,…,n, and the coincidence value is 0.877 for some value between 29 and 30. Finally, in case (7), the maximum coincidence value of 0.881 is achieved for some value between 77 and 78, where $a_{m}=\sum_{m=1}^{n}\left[\ln\left(n+1-m\right)/\left\{\sum_{r=1}^{n}\ln\left(n+1-r\right)\right\}\right]$ for all m=1,2,…,n. Overall, our analysis shows that the coincidence value is less than 8/9 (approximately 0.88) for all seven cases discussed in Table 2.

Coincidentally with the data from different scenarios, the inequality in those scenarios reaches a maximum point around 0.87 before dropping.

## 5. Real-World Data Indicating the Convergence of the Gini and Kolkata Indices in Various Socioeconomic Contexts

In this section, we investigate the emergence of pervasive inequality as an observable example of emergent properties in different socioeconomic systems. These systems exhibit universal characteristics of the the Gini (*g*) and Kolkata (*k*) inequality indices, as shown by real data collected from diverse social and economic systems. Our data collection and analysis were completed before the end of 2021. It is evident that these systems show emergent properties when their dynamics are not externally fine-tuned. In the current situation, this leads to an environment of unrestricted competition. To illustrate this phenomenon, we consider a range of socioeconomic systems, including income, income tax data, box office earnings from Hollywood (US) and Bollywood (India), daily Bitcoin price fluctuations, election candidates (vote shares), universities (excellence/citation sharing), publications by authors (citation shares), wars or social conflicts (human death shares), and sports (Olympic medal share), among others. These systems demonstrate the emergence of significant and widespread inequality indices, which we explore in detail in this section.

### 5.1. Socioeconomic Disparities: An Analysis of Income, Income Tax, and Box Office Earnings Data

**Figure 5:**
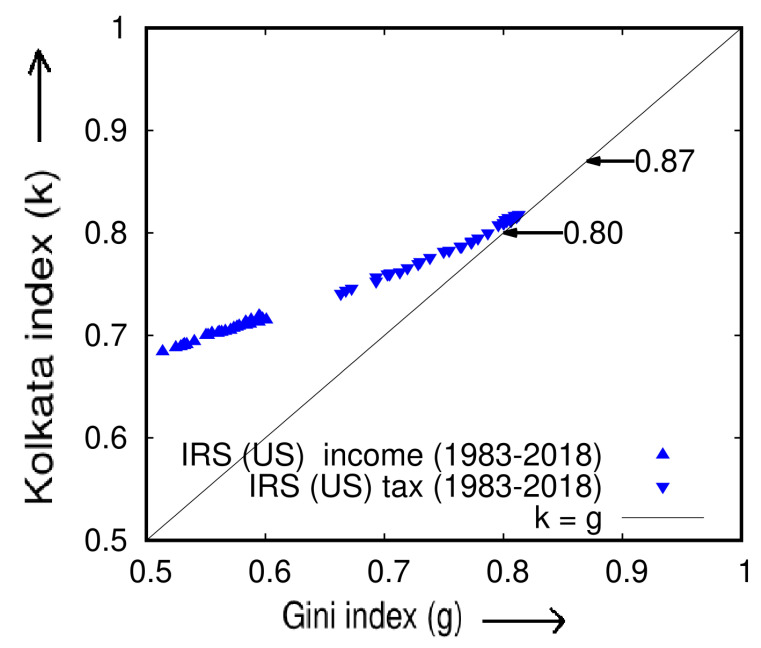
Plot of the Kolkata index (*k*) against the Gini index (*g*) for income and income tax data extracted from IRS (USA) data [19,20] from the years 1983 to 2018. The data were obtained from the corresponding Lorenz functions (L(p)) for each of these 36 years (adopted from [15]).

In a report by the United Nations Development Program [18], it was found that the global distribution of income is highly unequal, with the top 20% of the world’s population receiving 82.7% of the world’s income. Despite concerns that the top 1% of wealthy individuals possess 99% of the world’s wealth (as in ‘Occupy Wall Street’ protests), this observation suggests that 82% of the world’s wealth is actually owned by 18% of the population. To further explore this issue, we analyzed data from the IRS (US) [19,20] on the cumulative income of the poorest (*p*) fraction of people over a period of 36 years (1983–2018) and calculated the Lorenz function (L(p)). We also computed the Gini (*g*) and Kolkata (*k*) indices for each year (see Figure 5).

**Figure 6:**
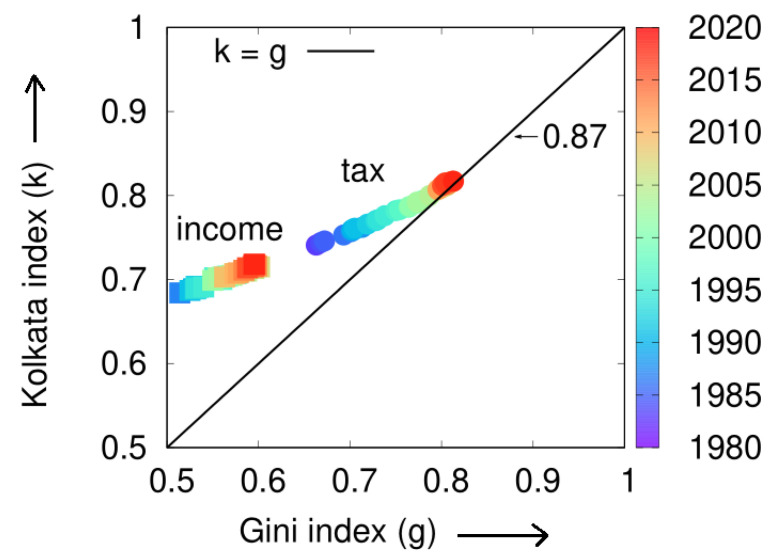
Trend of the Gini (*g*) and Kolkata (*k*) indices over time (year) for the US economy using IRS data [19,20]. The graph clearly shows an increasing trend in the inequality measures over time, indicating a decline in public welfare and a shift towards an SOC state of unrestricted competition. The value of *k* in the tax data, which is argued to be a better indicator of the prevailing inequality status, surpasses the Pareto value of 0.80 and is predicted to reach 0.87, similar to other socioeconomic systems (e.g., movie income or citations) in which public welfare programs are completely absent (this figure is adopted from [15]).

We present a visual representation of the analysis conducted on the IRS income and tax data [19,20] for a period of 36 years (1983–2018) in Figure 6. The graph depicts a consistent increase in the inequality indices (*g* and *k*) over the years, which converge towards a value of 0.87.

**Figure 7:**
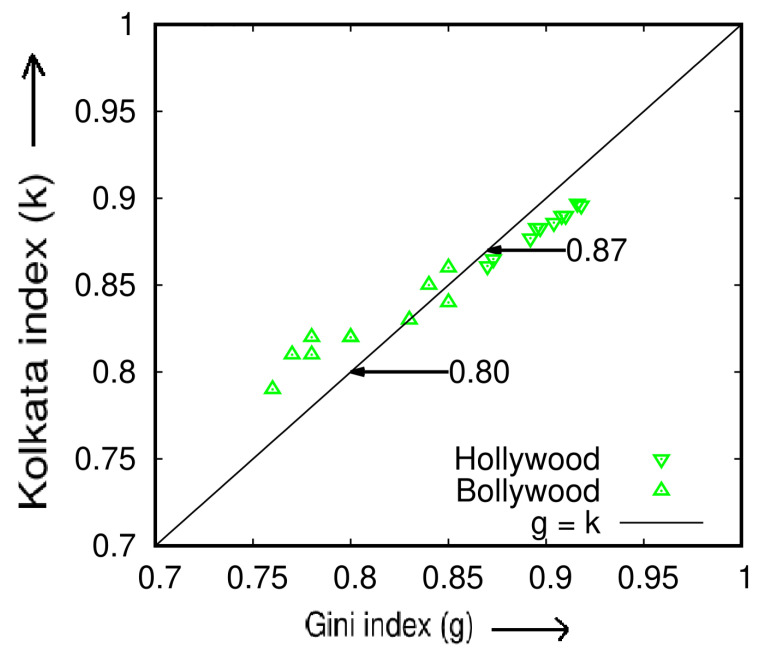
Scatter plot of the Kolkata index (*k*) versus the Gini index (*g*) for box office income obtained from Hollywood (USA, data source: [21]) and Bollywood (India, data source: [22]) over a period of 9 years from 2011 to 2019. The plot provides a comparative analysis of the inequality measures for these two major film industries (adopted from [15]).

Similarly, we extend our analysis to the yearly income generated by the film industry in Hollywood (USA [21]) and Bollywood (India [22]) for a period of 9 years (2011–2019). Table 3 and Figure 7 demonstrate the results of our analysis of the income data for these two film industries and show that both in Hollywood and in Bollywood, the box-office income inequality index (*k*) increases, on average, to 0.88 and 0.83, respectively.

In this case, we show that almost 88% of box office income share comes from only 12% of Hollywood movies measured from 2011 to 2019. Similarly, almost 83% of box office income share comes from only 17% of Bollywood movies measured from 2011 to 2019.

### 5.2. Inequality in Bitcoin Value Fluctuations: A Data Analysis Study

Bitcoin is a decentralized cryptocurrency that operates on a ledger system without any central bank control. Its introduction in 2008 and adoption in 2009 have made it the first and largest cryptocurrency, with a market value surpassing USD 1.03 trillionas of November 2021, accounting for 2.9% of the world’s total narrow money supply. While its decentralized nature has made it susceptible to market volatility, Bitcoin serves as an example of unrestricted competition in currency markets.

**Figure 8:**
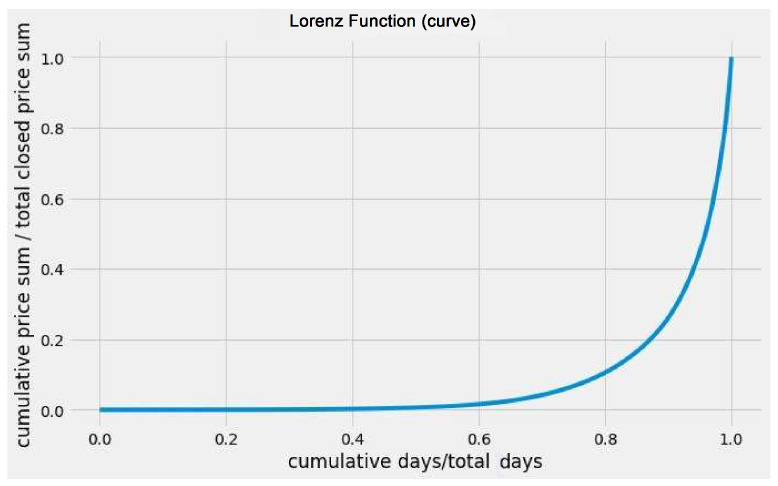
The Lorenz function (curve) depicts the distribution of the difference in the closing price of Bitcoin for consecutive days (adopted from [15]).

**Figure 9:**
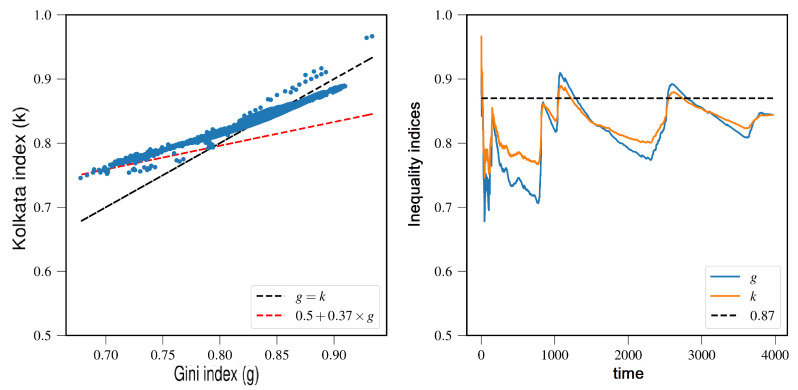
(**Left**): A graphical representation of the Kolkata index (*k*) plotted against the Gini index (*g*) for the statistical analysis of the daily Bitcoin price. (**Right**): temporal variation of the *g* and *k* indices. For comparison, a reference value of approximately 0.87 is provided in the figure (adopted from [15]).

In this study, we analyze the fluctuation of Bitcoin’s value, which is measured in USD, by collecting daily price data from 1 January 2010 to 24 November 2021, using data obtained from [23]. To investigate its value fluctuation, we calculate the absolute value of the price changes in consecutive days and collect the fractional closing price changes, except for a few days up to a date *t*(>$t_{0})$. We then generate the Lorenz curve (refer to Figure 8) for the closing price data up to a time (*t*(>>$t_{0})$) and proceed to estimate the *g* and *k* indices as discussed in Figure 9.

Figure 9 demonstrates that *g* and *k* exhibit a pattern of repeatedly approaching each other near the value of 0.87.

### 5.3. Inequality Analysis of Vote Data for Election Contestants

In this subsection, we analyze the inequality of vote shares among the candidates in the Indian parliamentary elections held in 2014 and 2019. Table 4 demonstrates that there exists a high level of inequality in the distribution of vote shares. The Gini and Kolkata indices were found to be 0.83 and 0.86 for the 2014 election and 0.85 and 0.88 for the 2019 election, respectively. The values of these indices are similar to the value of approximately 0.87 observed in the case of unrestricted competition.

### 5.4. Inequality Analysis for Citation Data of Different Journals and Universities

Data obtained from the ISI Web of Science [26] show that the citations of the papers published from different leading universities or institutions and leading journals are also unequal; here, we take the average value from 1980 to 2010 (see Table 5, Table 6 and Table 7). Table 8 shows that the most successful 18–25% of papers published by different universities or institutes and journals received 82–76% of citations.

**Figure 10:**
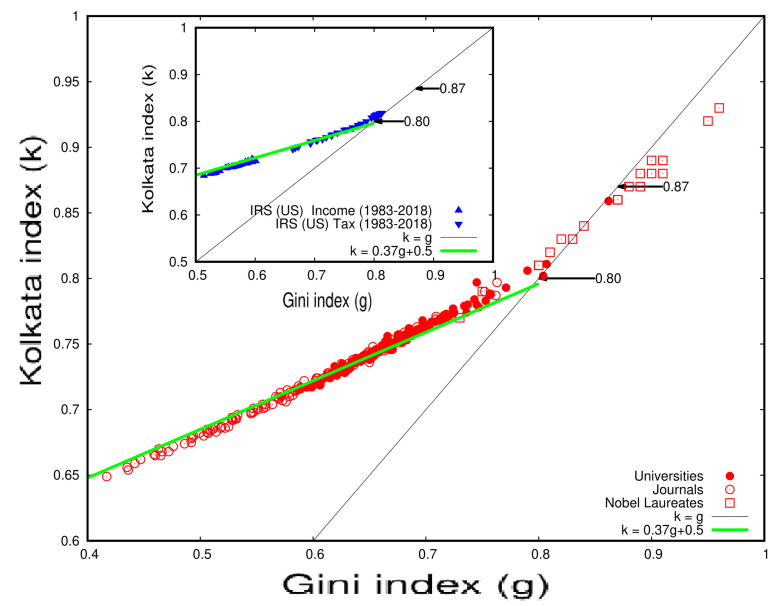
Comparison of the Gini index (*g*) and Kolkata index (*k*) obtained from the analysis of IRS (US) data on income, income tax, and income from movies from 1983 to 2018 (see inset in the figure) and citations of papers published by scientists from universities or institutes; published in journals; and by Nobel laureates in physics, chemistry, medicine, and economics (data taken from Refs. [27,28]). The initial variation of *k* against *g* for both income and income tax and for citations by universities, journals, and individual scientists is remarkably similar, showing quantitative agreement. The main figure illustrates this comparison (adopted from [15]).

Here, we compare the growth pattern of income inequality in the IRS data (USA) with citation inequality for papers published by established universities (see Table 5 and Table 6) published in established journals (see Table 7) and by individual Nobel laureate scientists (see Table 9). The data for these comparisons were taken from other publications (Refs. [26,27,28]). Figure 10 displays the results, which show that the *k* and *g* inequality indices of all these categories drift linearly toward a universal value of k=g≃0.87 under unrestricted competition. This suggests that approximately 87% of the wealth, citations, or votes are possessed, earned, or won by 13% of people, papers, or election candidates, respectively.

### 5.5. A Study of Inequality in Citations: An Analysis of Individual Authors and Award Recipients

In this section, we present two tables with statistical analysis of research papers and their citations for 20 distinguished scientists who have won Nobel Prizes in economics, physics, chemistry, and biology/physiology/medicine, as well as for several international prize winners in mathematics and physics. Table 9 shows the analysis for Nobel laureates, while Table 10 presents the results for the international prize winners. The data were collected from Google Scholar during the first week of January 2021, and the names of the scientists are reported in the same format as they appear on their respective Google Scholar pages.

**Figure 11:**
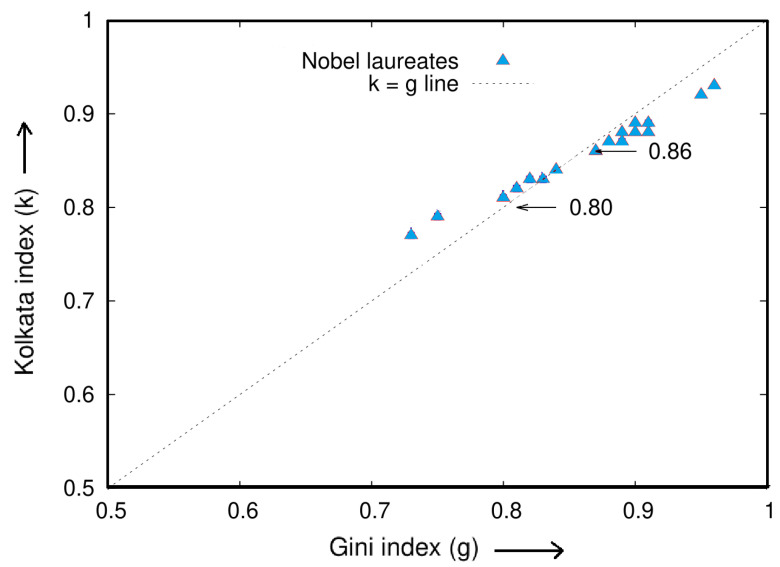
Plot of the values of the Kolkata (*k*) index versus the corresponding Gini (*g*) index for the citation statistics of publications by 20 selected Nobel laureates, as shown in Table 9. The plot suggests a coincidence value of k=g=0.86±0.06, as adapted from a previous study [27].

**Figure 12:**
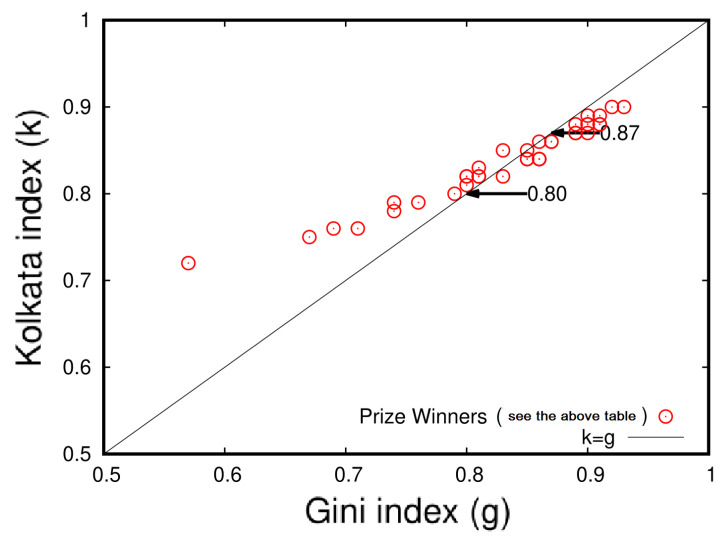
Plot of the Kolkata (*k*) index versus the Gini (*g*) index for the citation inequalities in papers published by individual prize winners. The data were extracted from the corresponding Lorenz function (L(p)) for each scientist and are presented in Table 10 (adopted from [15]).

Figure 11 and Figure 12 illustrate the inequality analysis for the 20 Nobel laureates and prize winners. These figures demonstrate an ideal example of the dynamics of wealth inequality without any external interventions or fine tuning. Furthermore, the Gini index (*g*) and the Kolkata index (*k*) approach each other at a value of approximately 0.87 in both cases. This indicates that the results are consistent and robust across groups of distinguished scientists.

In Figure 11 and Figure 12, we see that an average 15% of the papers published by successful individual authors received about 85% of his/her total citations. The Gini and *k* indices coincide around ≃0.87.

### 5.6. Similarity in the Behavior of the Gini and Kolkata Indices across Multiple Domains: A Universality Study

**Figure 13:**
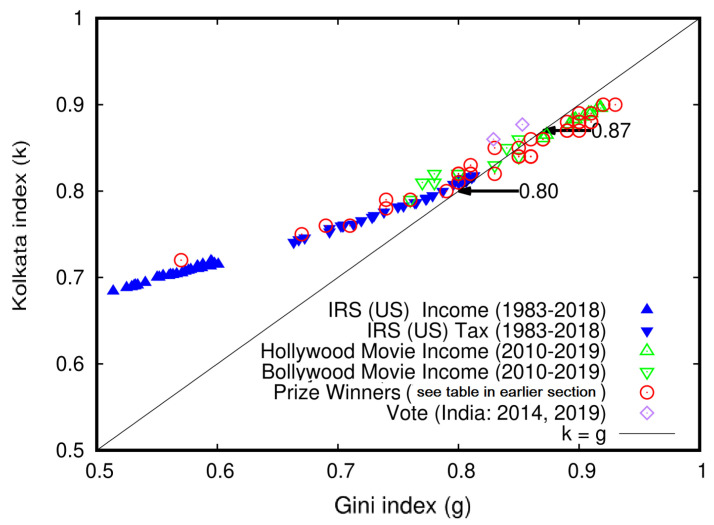
A compiled plot of the Kolkata index (*k*) values versus corresponding Gini index (*g*) values for several cases analyzed in previous subsections, including household income and income tax data (Figure 5, Figure 6), movie income (Figure 7), citation inequalities among individual prize winners (Table 10, Figure 12), and vote share inequalities among election contestants (Table 4). The results suggest that there may be universal inequality measures across social institutions, as the data points in the plot converge towards a common value of k=g=0.87±0.02. This observation has important implications for understanding the nature and extent of wealth inequality across different domains of society (adopted from [15]).

In this section, we aim to consolidate the findings on the Gini (*g*) and Kolkata (*k*) indices from previous subsections. We gathered estimates of *g* and *k* from various sources, including IRS (US) data [19,20] on household income and income tax spanning 1983–2018; the citation data from papers published by 40 international prize winners (Fields medalists, ASICTP Dirac medalists, Boltzmann medalists, and von Neumann awardees), as shown in Table 10; and the vote share data from the Indian parliamentary elections in 2014 and 2019, displayed in Table 4. We present the compiled results in Figure 13. The collective analysis of all these results reveals a universal trend of inequality growth across various social institutions, markets (income and wealth), academic institutions (citations), and elections (vote shares among the candidates). Furthermore, the analysis shows that the measures of inequality converge to k=g=0.87±0.02.

### 5.7. Inequality Analysis for Manmade Conflicts and Natural Disasters

With respect to manmade conflicts such as war, battle, armed conflict, terrorism, murder, etc., an average of 85% of human deaths are caused by 15% of social conflicts, including war (see Table 11).

We also show that for natural disasters such as earthquakes, floods, tsunamis, etc., almost 95% of human deaths are caused by 5% of disasters (see Table 12).

### 5.8. Inequality Analysis in Computing Systems

The field of computer science has long recognized the adage that “20% of the code contains 80% of the errors”, as noted in [30]. Consequently, software developers have a vested interest in identifying and rectifying this critical 20% of the codebase in order to enhance the quality of the software. In a related finding, researchers have also observed that approximately 80% of the functionality of a given software program can typically be implemented in just 20% of the total development time. Conversely, the remaining 20% of the software’s features, which represent the most challenging and time-consuming aspects of the coding process, often require the remaining 80% of the total development time. This factor is commonly taken into account when estimating the cost and timeline for software development using the constructive cost model (COCOMO) approach. Therefore, in computing, we see that 20% of the code contains 80% of the errors.

### 5.9. Inequality Analysis for Sports: Olympic Medal Share

In recent discussions, scholars have posited that the concept of inequality also applies to the field of sports, where a few top performers often dominate the majority of victories. This phenomenon is exemplified in the sport of baseball, where wins above replacement (WAR) is used as a composite metric to gauge a player’s overall importance to a team. Recent statistical analyses have revealed that a mere 15% of baseball players accounted for 85% of the total wins, while the remaining 85% of players were responsible for generating only 15% of the wins [31]. These findings provide compelling evidence for the existence of significant inequality within the sport, indicating that a small minority of players drive the majority of team successes. The inequality statistics (reflected in the Gini and Kolkta index values) for the country-wise inequalities in Olympic medal wins are shown in Table 13. Typically, 13 to 16 percent of countries win 84 to 87 percent of Olympic medals (gold, silver, or bronze) in the Summer Olympics (see Table 13; statistics for the last four Olympics from ref. [32]).

## 6. Growing Avalanche Size Inequalities in Sand Pile Models: Universality near the SOC Point

All the observations reported so far indicate that the *k* index reaches a critical value of k=g before the inequality falls again. Such an observation implies that the k=g point can be characterized as a critical point for society. Further observations of physical self-organized critical systems indicate the same kind of behavioral pattern [16]. As all of the above observations were made for unrestricted competitive scenarios in which only the winners obtain all the facilities, we can expect that in a real society, such a point exists and that inequality can never exceed that point because of government subsidies that aid the poor population.

According to the self-organized criticality (SOC) framework, the critical point is an attractor; we found that (see, for example, [16]) immediately preceding the SOC point, the avalanche size inequality attains a value approximately equal to 0.86. This observation is consistent with our observations discussed in the previous sections.

**Figure 14:**
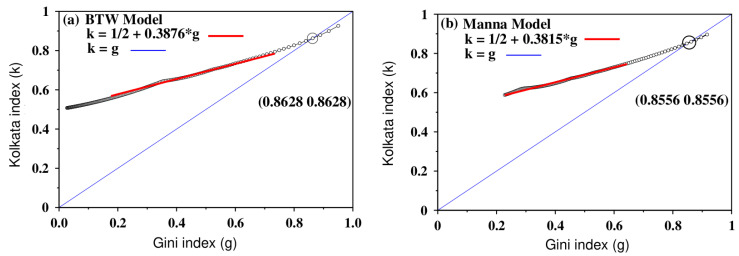
The relationship of the *k* index versus the Gini index (*g*) for two sand pile models: (**a**) the BTW model and (**b**) the Manna model. In both cases, the initial portions of the curves follow a straight line with slightly different slopes, as demonstrated in the figures. The crossing points of the curves with the line g=k are 0.8628 and 0.8556 for the BTW and Manna models, respectively. This figure was adapted from [16].

Here, we present the results of a comparison of the *k* and *g* indices for two different sand pile models, namely the Bak–Tang–Wiesenseld (BTW) model and the Manna model [16]. Figure 14 illustrates the *k* versus *g* relationship for these models, with panel (a) showing the results for the BTW model and panel (b) showing the results for the Manna model. The plots depict a linear relationship between *k* and *g* for the initial part of the curve. For the BTW model, the slope of the linear fit is 0.3876, while for the Manna model, the slope is slightly lower, at 0.3815. The intersection of the plots with the line of equality (k=g) occurs at 0.8628 and 0.8556 for the BTW and Manna models, respectively. It is important to note that these results were obtained for a system size of L×L=512×512.

**Figure 15:**
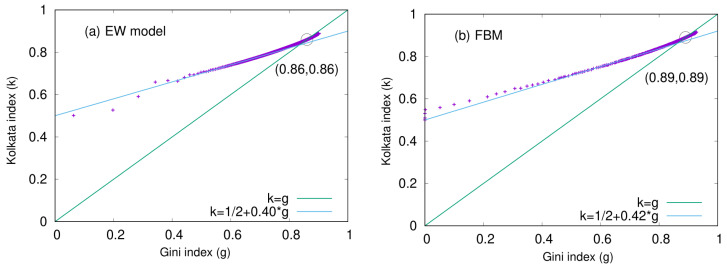
The relationships between the *k* index and Gini index (*g*) for the EW and centrally loaded fiber bundle models. Figure (**a**) shows the *k* versus *g* plot for the EW model, with an initial slope of 0.40. Figure (**b**) displays the same plot for the centrally loaded fiber bundle model, with an initial slope of 0.42. The figure is adapted from [16].

The relationship between the Kolkata index (*k*) and the Gini index (*g*) is also analyzed here for two other SOC models, namely the Edwards–Wilkinson (EW) model and the fiber bundle model (FBM) [16]. In Figure 15a, the *k* values are plotted against their corresponding *g* values for the EW model. A linear relationship is observed in the initial section of the plot, with a slope of 0.40. Similarly, in Figure 15b, the *k* versus *g* plot is shown for the centrally loaded fiber bundle model. In this case, the initial linear slope is measured to be 0.42. These results suggest that the *k* index and Gini index are linearly related in the early stages of both models, with slightly different slopes.

In the several SOC models considered here, we observe a remarkably consistent coincidence of the avalanche size inequality indices (*g* and *k*) at around g=k≃0.86 immediately preceding the arrival of the SOC point. This is also consistent our previous observations in the inequality measures in various socioeconomic contexts. We consider this to be noteworthy.

## 7. Summary and Discussion

Disparities between social classes are constantly present (see, e.g., [33,34] for some recent discussions), which has been proposed that it is an emergent trait of complex socioeconomic systems [35] with many interacting parts. It may be mentioned at this point that allowing for a higher probability of exchange for the poor in conservative kinetic exchange models induces (see, e.g., [36,37,38]), in a novel, self-organized way, a minimum “poverty level”, thereby reducing inequality. Herein, we attempted to show that the extreme social inequality we find in society is the result of built-in self-organized critical dynamics. A self-organized criticality [11,39] framework has been proposed to explain the evolutionary behavior of diverse systems, such as wealth distributions, financial markets, cryptocurrencies, citation dynamics, etc. However, a major unresolved issue concerns the appropriate quantification of the observed disparities and their potential universality across systems. Pareto’s 80/20 law, which implies that 80% of the wealth ends up in the hands of 20% of the richest (k=0.80) members of society, has traditionally been used as a benchmark for measuring the degree of extreme social inequality. Moreover, we attempted to determine the amount of social and economic inequality in a number of very competitive systems without any outside interventions to stop halt inequality among the agents. This self-tuning feature of competitive dynamics in various social sectors suggests similar inequality behavior in the SOC system. Herein, we studied a significant number of socioeconomic systems through the framework of SOC architecture. This approach has been used to analyze a range of systems, including financial markets [40], citation evolution [39,41], cryptocurrencies [42], and political behavior [43], among others. Despite the diversity of these systems, our analysis reveals that the behavior of inequality indices, particularly the Gini (*g*) and Kolkata (*k*) indices, demonstrates near-universal characteristics across socioeconomic systems. Specifically, we observe that these indices tend to converge towards a value of approximately 0.87. This finding is particularly noteworthy, given that similar behavior has been observed in SOC models of physical systems [16,44]. A recent research publication [45] presented findings that highlight the high level of inequality in group size distribution. The authors reported a *g* index value of 0.90 for both the theoretical and empirical observations of group size distribution. We note the consistency of such a high value of the *g* index with our observations.

In Section 2, we presented a chronological account of the development of social inequality measures since 1896 and demonstrated their similarity to those in sand pile models immediately prior to their respective self-organized critical (SOC) points. In Section 3, we discussed the Lorenz curve, the Gini (*g*) index, and the Kolkata (*k*) index in detail and presented proofs of their properties, as well as exemplary calculations. In Section 4 of our review, we conducted an investigation into several analytical characteristics of the Lorenz function (L(p)). Our analysis, supported by Table 1 and Table 2, led us to the conclusion that for a wide range of plausible analytic forms of L(p), the values of the Gini (*g*) and Kolkata (*k*) indices that correspond to coincidence points fall within the range of 4/5 (equivalent to 0.80) to 8/9 (equivalent to 0.88⋯). This finding suggests that the values of *g* and *k* are tightly constrained and are influenced by the analytic properties of the Lorenz function (L(p)). In particular, in Section 5, we considered datasets accounting for factors such as earnings from different sectors, Bitcoin price fluctuations, citations of selected prize-winning scientists, and votes received by various candidates in elections. We conducted an analysis of income data from multiple sources. Specifically, we examined data from the Internal Revenue Service (IRS) in the United States, as well as income tax data, over a period of 36 years spanning 1983 to 2018. The IRS data were obtained from previous research [19,20]. Additionally, we investigated income data from the Hollywood movie industry in the United States, which was sourced from a prior study [21], and data from the Bollywood movie industry in India [22] for the time period ranging from 2011 to 2019. These data sources were analyzed in detail in Section 5.1 of our study. In Section 5.2, we showed data for Bitcoin price fluctuations [23], and in Section 5.3, we examined the vote share data pertaining to candidates who ran in parliamentary elections in India during in 2014 and 2019. We conducted an analysis of this data with the aim of exploring inequalities in the vote share among candidates [24,25]. In this research, we present data on the citations of published papers from several prominent universities and institutions, as well as leading journals, which can be found in Section 5.4 and are cited in [7]. We also conducted an analysis of citation data from Google Scholar for 20 selected individuals who have been awarded Nobel Prizes in the fields of economics, physics, chemistry, and biology/physiology/medicine, as well as individuals who have been awarded the Fields Medal (mathematics), the Boltzmann Medal (physics),the ASICTP Dirac Medal (physics), or the John von Neumann Award (social science) in various years. We focused on individuals who have their own Google Scholar pages with “verified email” addresses; the results of our analysis are presented in Section 5.5. We utilized these data sources to investigate sectoral inequality and computed the associated Gini (*g*) and Kolkata (*k*) indices. The results of our analysis are presented in Figure 5, Figure 7 and Figure 9, Figure 10, Figure 11 and Figure 12 and summarized in Table 3, Table 4, Table 5, Table 6, Table 7, Table 8, Table 9 and Table 10. We also compiled these findings into a single figure, shown in Section 5.6 as Figure 13. Our analysis revealed a universal value of approximately 0.87 for the coinciding *g* and *k* indices, indicating an emerging trend of increasing disparities under conditions of competition. Moreover, in Section 5.7, we investigated a similar trend for manmade conflicts such as war, terrorism, etc., as well as for natural disasters such as earthquakes, tsunamis, etc. (see Table 11 and Table 12). In Section 5.8 and Section 5.9, we discussed the universality of the *k* index for computing systems and sports, respectively. The results of our empirical investigation reveal a consistent pattern in the dynamical behavior of the Kolkata index (*k*) and the Gini index (*g*) across various scenarios. Specifically, our findings indicate that these inequality measures converge towards a universal value of k=g=0.87±0.02 in situations in which competitions are not subject to any restrictions. This trend was observed consistently across all socioeconomic systems, highlighting the robustness and universality of this phenomenon. When we talk about dynamics, we are referring to the long-term changes and eventual saturation brought about in the aforementioned systems. We presented a graphical representation of the *g* and *k* indices for the daily price fluctuations of Bitcoin over the period of a decade from 2010 to 2021. As shown in Figure 9, our findings suggest that the *g* and *k* indices tend to stabilize at a value of approximately 0.87, which lends further support to our conclusions. This pattern suggests that without the intervention of a central bank, such as in the case of national currencies, the inequality indices for cryptocurrencies converge. Specifically, both *g* and *k* approach a value of 0.87 before decreasing. Our results indicate that the daily swings in the price of Bitcoin, on average, do not exceed this limiting value (g=k≃0.87). Table 13 (Section 5.9 on Inequality Analysis for sports: Olympic medal share) shows that the *k* index value typically ranges from 0.84 to 0.87, implying that 13 to 16 percent of countries win 84 to 87 percent of Olympic medals.

We investigated the behavior of inequality indices over time and found that they have not yet converged to the predicted attractor value of 0.87 that results from a self-organized critical (SOC) state. However, our analysis presented in Figure 6 in Section 5.1 reveals that both the Gini index (*g*) and the Kolkata index (*k*) have exhibited a steady increase over time. This trend is likely attributable to the gradual reduction in public welfare programs in the United States. Interestingly, our results demonstrate that the Pareto value of k=0.80 has already been surpassed. It is possible to estimate that it will reach 0.87 if all of the aforementioned public assistance programs are eliminated, thereby allowing participants to enter a state of unrestricted competition. In Section 6, we discussed the SOC state of different physical systems (such as the BTW model and Manna model) and calculated their inequality indices (*g* and *k*) in terms of their growing avalanche sizes, showing the universality trend, i.e., g=k≃0.86 (Figure 14 and Figure 15).

The social dynamics of competition take the index values of g=k≃0.87, indicating that roughly 87% of wealth, citations, votes, or Olympic medals are possessed, earned, or won by 13% of the population, papers, election candidates, or (Olympic participant) countries, respectively in cases of unrestricted competition in which no welfare support towards equality is available. This may be a quantitative and universal (across all social sectors) version of the 80/20 law (k=0.80) observed by Pareto more than a century ago. This property of the inequality indices is intrinsic to the SOC character of the underlying dynamics, and it has been demonstrated to be present in a wide variety of SOC models in physical science [16,44].

Previous studies [17,46] established that the Gini index (*g*) can be considered to represent the information entropy of social systems, while the Kolkata index (*k*) can be thought of as a representation of the inverse of the effective temperature of such systems. An increase in *k* corresponds to a decrease in the average wealth of a society in circulation, resulting in a decrease in temperature. In this study, we observed that the ratio of g/k, which is equivalent to free energy, displays an identical value at multiple points (g=k≃0.87 and g=k=1). These findings suggest the existence of a first-order-like phase transition [28] at the point at which g=k≃0.87. This reinforces the idea that the relationship between the Gini and Kolkata indices can be analyzed from a thermodynamic perspective. This inequality growth is entropy-driven, as conjectured in the context of self-organized sand pile systems (see, e.g., [47,48]), similar to those explored herein.

## Figures and Tables

**Table 1:** The set of Lorenz functions of the form $L\left(p\right)=p^{n}$ with *p* ranging within the interval [0,1] and *n* values ranging from 1 to 20.

*n*	$L\left(p\right)=p^{n}$	$g_{n}$	$k_{n}$
(n=1)	*p*	0	$\frac{1}{2}$
(n=2)	$p^{2}$	$\frac{1}{3}$	$\frac{\sqrt{5}-1}{2}≃0.618$
(n=3)	$p^{3}$	$\frac{1}{2}$	0.682
(n=4)	$p^{4}$	$\frac{3}{5}$	0.725
(n=5)	$p^{5}$	0.667	0.755
(n=6)	$p^{6}$	0.714	0.778
(n=7)	$p^{7}$	0.750	0.797
(n=8)	$p^{8}$	0.778	0.812
(n=9)	$p^{9}$	0.800	0.824
(n=10)	$p^{10}$	0.818	0.835
(n=11)	$p^{11}$	0.833	0.844
(n=12)	$p^{12}$	0.846	0.853
(n=13)	$p^{13}$	0.857	0.860
(n=14)	$p^{14}$	0.867	0.866
(n=15)	$p^{15}$	0.875	0.872
(n=16)	$p^{16}$	0.882	0.877
(n=17)	$p^{17}$	0.889	0.882
(n=18)	$p^{18}$	0.895	0.886
(n=19)	$p^{19}$	0.900	0.890
(n=20)	$p^{20}$	0.905	0.894

**Table 2:** Different polynomial Lorenz functions (adopted from Banerjee et al. [15]).

*Case*	$L_{\left(n,a\right)}\left(p\right)$	$g_{\left(n,a\right)}=k_{\left(n,a\right)}$	Intervalofn
(1)	$p^{n}$	0.865	(13, 14)
(2)	$\sum_{m=1}^{n}\left(\frac{1}{n}\right)p^{m}$	0.869	(65, 66)
(3)	$\left\{\frac{\left(n-1\right)}{n}\right\}p+\;\sum_{m=2}^{n}\left\{\frac{1}{n(n-1)}\right\}p^{m}$	Impossibility	–
(4)	$\sum_{m=1}^{n-1}\left\{\frac{1}{n(n-1)}\right\}p^{m}+\;\left\{\frac{\left(n-1\right)}{n}\right\}p^{n}$	0.874	(17, 18)
(5)	$\sum_{m=1}^{n}\left\{\frac{6m(n+1-m)}{n(n+1)(n+2)}\right\}p^{m}$	0.874	(40, 41)
(6)	$\sum_{m=1}^{n}\left\{\frac{2(m+1)}{n(n+3)}\right\}p^{m}$	0.877	(29, 30)
(7)	$\sum_{m=1}^{n}\left\{\frac{\ln(n+1-m)}{\sum_{r=1}^{n}\ln\left(n+1-r\right)}\right\}p^{m}$	0.881	(77, 78)

**Table 3:** An analysis of income inequality in box office earnings for movies released during the period of 2011–2019 in two major film industries, Hollywood (USA) and Bollywood (India). Data taken from refs. [21,22].

Movie	Box Office Collection from Hollywood (USA) Movies		
Release Year	Total Movies	Gini (*g*)	Kolkata (*k*)
2010	651	0.87	0.86
2011	730	0.87	0.87
2012	807	0.89	0.88
2013	826	0.90	0.88
2014	849	0.90	0.88
2015	847	0.91	0.89
2016	856	0.90	0.89
2017	852	0.91	0.89
2018	993	0.92	0.90
2019	911	0.92	0.90
**Movie**	**Box Office Collection** **from Bollywood (India) Movies**		
**Release** **Year**	**Total** **Movies**	**Gini** **(** ***g*** **)**	**Kolkata** **(** ***k*** **)**
2010	139	0.77	0.81
2011	123	0.78	0.82
2012	132	0.78	0.81
2013	136	0.76	0.79
2014	145	0.8	0.82
2015	166	0.8	0.82
2016	215	0.83	0.83
2017	251	0.85	0.84
2018	218	0.84	0.85
2019	246	0.85	0.86

**Table 4:** Gini (*g*) and Kolkata (*k*) index values obtained from the Lorenz function (L(p)) for the vote shares in the Indian parliamentary elections held in the years 2014 and 2019, where the number of contesting candidates was over 8000 in each year. The data used for the analysis were obtained from references [24,25].

Year	Total Voters	*g*	*k*
2014	$5\times 10^{8}$	0.83	0.86
2019	$6\times 10^{8}$	0.85	0.88

**Table 5:** Gini and Kolkata indices for citation inequalities of publications by authors from selected universities analyzed in December 2013 using data obtained from the ISI Web of Science (adapted from [7,27]).

Inst./Univ.	Year	ISI Web of Science Data			
		$N_{p}$	$N_{c}$	Index Values	
				g	k
Melbourne	1980	866	16,107	0.67	0.75
	1990	1131	30,349	0.68	0.75
	2000	2116	57,871	0.65	0.74
	2010	5255	63,151	0.68	0.75
Tokyo	1980	2871	60,682	0.69	0.76
	1990	4196	108,127	0.68	0.76
	2000	7955	221,323	0.70	0.76
	2010	9154	91,349	0.70	0.76
Harvard	1980	4897	225,626	0.73	0.78
	1990	6036	387,244	0.73	0.78
	2000	9566	571,666	0.71	0.77
	2010	15,079	263,600	0.69	0.76
MIT	1980	2414	101,929	0.76	0.79
	1990	2873	156,707	0.73	0.78
	2000	3532	206,165	0.74	0.78
	2010	5470	109,995	0.69	0.76
Cambridge	1980	1678	62,981	0.74	0.78
	1990	2616	111,818	0.74	0.78
	2000	4899	196,250	0.71	0.77
	2010	6443	108,864	0.70	0.76
Oxford	1980	1241	39,392	0.70	0.77
	1990	2147	83,937	0.73	0.78
	2000	4073	191,096	0.72	0.77
	2010	6863	114,657	0.71	0.76

**Table 6:** Gini and Kolkata indices for citation inequalities of publications by authors from selected Indian universities/institutes analyzed in December 2013 using data obtained from the ISI Web of Science (adapted from [7]).

Inst./Univ.	Year	ISI Web of Science Data			
		$N_{p}$	$N_{c}$	Index Values	
				g	k
SINP	1980	32	170	0.72	0.74
	1990	91	666	0.66	0.73
	2000	148	2225	0.77	0.79
	2010	238	1896	0.71	0.76
IISC	1980	450	4728	0.73	0.78
	1990	573	8410	0.70	0.76
	2000	874	19,167	0.67	0.75
	2010	1624	11,497	0.62	0.73
TIFR	1980	167	2024	0.70	0.76
	1990	303	4961	0.73	0.77
	2000	439	11,275	0.74	0.77
	2010	573	9988	0.78	0.79
Calcutta	1980	162	749	0.74	0.78
	1990	217	1511	0.64	0.74
	2000	173	2073	0.68	0.74
	2010	432	2470	0.61	0.73
Delhi	1980	426	2614	0.67	0.75
	1990	247	2252	0.68	0.76
	2000	301	3791	0.68	0.76
	2010	914	6896	0.66	0.74
Madras	1980	193	1317	0.69	0.76
	1990	158	1044	0.68	0.76
	2000	188	2177	0.64	0.73
	2010	348	2268	0.78	0.79

**Table 7:** Gini and Kolkata indices for citation inequalities of publications in selected science journals in December 2013 using data obtained from the ISI Web of Science (adapted from [7]).

Inst./Univ.	Year	ISI Web of Science Data			
		$N_{p}$	$N_{c}$	Index Values	
				g	k
Nature	1980	2904	178,927	0.80	0.81
	1990	3676	307,545	0.86	0.85
	2000	3021	393,521	0.81	0.82
	2010	2577	100,808	0.79	0.81
Science	1980	1722	111,737	0.77	0.80
	1990	2449	228,121	0.84	0.84
	2000	2590	301,093	0.81	0.82
	2010	2439	85,879	0.76	0.79
PNAS(USA)	1980	-	-	-	-
	1990	2133	282,930	0.54	0.70
	2000	2698	315,684	0.49	0.68
	2010	4218	116,037	0.46	0.66
Cell	1980	394	72,676	0.54	0.70
	1990	516	169,868	0.50	0.68
	2000	351	110,602	0.56	0.70
	2010	573	32,485	0.68	0.75
PRL	1980	1196	87,773	0.66	0.74
	1990	1904	156,722	0.63	0.74
	2000	3124	225,591	0.59	0.72
	2010	3350	73,917	0.51	0.68
PRA	1980	639	24,802	0.61	0.73
	1990	1922	54,511	0.61	0.72
	2000	1410	38,948	0.60	0.72
	2010	2934	26,314	0.53	0.69
PRB	1980	1413	62,741	0.65	0.74
	1990	3488	153,521	0.65	0.74
	2000	4814	155,172	0.59	0.72
	2010	6207	70,612	0.53	0.69
PRC	1980	630	19,373	0.66	0.75
	1990	728	15,312	0.63	0.73
	2000	856	19,143	0.57	0.71
	2010	1061	11,764	0.56	0.70
PRD	1980	800	36,263	0.76	0.80
	1990	1049	33,257	0.68	0.76
	2000	2061	66,408	0.61	0.73
	2010	3012	40,167	0.54	0.69
PRE	1980	-	-	-	-
	1990	-	-	-	-
	2000	2078	51,860	0.58	0.71
	2010	2381	16,605	0.50	0.68

**Table 8:** Average citation share of papers published from 1980 to 2010 by different universities/institutions and in journals [26].

Inst./Univ./Journ	Papers (%)	Citations (%)	Comments
Harvard (Univ)	22	78	About 23% of the papers
MIT (Univ)	22	78	published by leading
IISC (Inst)	25	75	universities/institutions received 77%
TIFR (Inst)	23	77	of the citations.
			About 19% of the papers
Nature (Journ)	18	82	published in leading
Science (Journ)	19	81	journals received 81% of
			the citations.

**Table 9:** Statistical analysis of research papers and their citations for 20 Nobel laureates in economics (Econ), physics (Phys), chemistry (Chem), and biology/physiology/medicine (Bio). The data were collected from their individual Google Scholar pages with a verifiable email site during the first week of January 2021. To be included in the analysis, the Laureates had to have at least 100 entries (papers or documents), with the latest not before 2018. These Nobel laureates have published a range of papers, from 111 to 3000, with $N_{p}$ denoting the number of papers. The laureates’ names appear in the same form as they do on their respective Google Scholar pages (adopted from Ghosh et al. [28]).

Award	Name of Recipient	Google Scholar Citation Data			
		$N_{p}$	$N_{c}$	Index Values	
				g	k
NOBEL Prize (Econ.)	Joseph E. Stiglitz	3000	323,473	0.90	0.88
	William Nordhaus	783	74,369	0.87	0.86
	Abhijit Banerjee	578	59,704	0.89	0.88
	Esther Duflo	565	69,843	0.91	0.89
	Paul Milgrom	365	102,043	0.90	0.89
	Paul Romer	255	95,402	0.96	0.93
NOBEL Prize (Phys.)	Hiroshi Amano	1300	44,329	0.80	0.81
	David Wineland	720	63,922	0.88	0.87
	Gérard Mourou	700	49,759	0.82	0.83
	Serge Haroche	533	40,034	0.87	0.86
	A. B. McDonald	492	20,346	0.91	0.88
	David-Thouless	273	47,452	0.89	0.87
	F.D.M. Haldane	244	41,591	0.87	0.86
	Donna Strickland	111	10,370	0.95	0.92
NOBEL Prize (Chem.)	Joachim Frank	853	48,077	0.80	0.81
	Frances Arnold	682	56,101	0.75	0.79
	Jean Pierre Sauvage	713	57,439	0.73	0.77
	Richard henderson	245	27,558	0.84	0.84
NOBEL Prize (Bio.)	Gregg L. Semenza	712	156,236	0.81	0.82
	Michael Houghton	493	49,368	0.83	0.83

**Table 10:** Citation data analysis for selected prize winners in Physics, mathematics, and social sciences. The selected prize winners are recipients of the Dirac Medal, Boltzmann Medal, Fields Medal, and John von Neumann Award. The analysis includes only those prize winners who have verified email addresses and an updated Google Scholar page after 2018. The data were collected in the first week of April 2021. For each individual scientist, the table reports the total number of papers ($N_{p}$), total citations ($N_{c}$), Gini index (*g*), and Kolkata index (*k*).

Award	Name of Recipient	Google Scholar Citation Data			
		$N_{p}$	$N_{c}$	Index Values	
				g	k
FIELDS Medal (Math.)	Terence Tao	604	80,354	0.88	0.86
	Edward Witten	402	314,377	0.74	0.79
	Alessio Figalli	228	5338	0.67	0.75
	Vladimir Voevodsky	189	8554	0.83	0.85
	Martin Hairer	181	7585	0.74	0.78
	Andrei Okounkov	134	10,686	0.69	0.76
	Stanislav Smirnov	79	4144	0.76	0.79
	Richard E. Borcherds	61	5096	0.81	0.83
	Ngo Bao Chau	44	1214	0.71	0.76
	Maryam Mirzakhani	25	1769	0.57	0.74
ASICTP DIRAC Medal (Phys.)	Rashid Sunyaev	1789	103,493	0.91	0.88
	Peter Zoller	838	100,956	0.81	0.82
	Mikhail Shifman	784	52,572	0.85	0.84
	Subir Sachdev	725	58,692	0.83	0.82
	Xiao Gang Wen	432	46,294	0.8	0.82
	Alexei Starobinsky	328	47,359	0.81	0.82
	Pierre Ramond	318	23,610	0.89	0.87
	Charles H. Bennett	236	89,798	0.9	0.88
	V. Mukhanov	208	27,777	0.85	0.84
	M A Virasoro	150	12,886	0.9	0.87
BOLTZMANN Award (Stat. Phys.)	Elliott Lieb	755	76,188	0.86	0.85
	Daan Frenkel	736	66,522	0.8	0.81
	Harry Swinney	577	46,523	0.86	0.84
	Herbert Spohn	446	25,188	0.79	0.8
	Giovanni Gallavotti	446	15,583	0.86	0.84
JHON Von NEUMANN Award (Social Sc.)	Daron Acemoglu	1175	172,495	0.91	0.89
	Olivier Blanchard	1150	113,607	0.91	0.89
	Dani Rodrik	1118	136,897	0.9	0.89
	Jon Elster	885	79,869	0.89	0.87
	Jean Tirole	717	201,410	0.91	0.88
	Timothy Besley	632	57,178	0.89	0.88
	Maurice Obstfeld	586	73,483	0.9	0.88
	Alvin E. Roth	566	54,104	0.87	0.86
	Avinash Dixit	557	82,536	0.93	0.9
	Philippe Aghion	490	119,430	0.85	0.85
	Matthew O. Jackson	397	39,070	0.86	0.84
	Emmanuel Saez	310	48,136	0.86	0.86
	Mariana Mazzucato	236	12,123	0.87	0.86
	Glenn Loury	226	13,352	0.92	0.9
	Susan Athey	203	18,866	0.8	0.82

**Table 11:** Estimated Gini (*g*) and Kolkata (*k*) index values applied to manmade conflicts such as wars and acts of terrorism. Data were adapted from a previous study [29] and represent death counts as a measure of inequality.

Type of Conflict	*g* Index	*k* Index
War	0.83 ± 0.02	0.85 ± 0.02
Battle	0.82 ± 0.02	0.85 ± 0.02
Armed conflict	0.85 ± 0.02	0.87 ± 0.02
Terrorism	0.80 ± 0.03	0.83 ± 0.02
Murder	0.66 ± 0.02	0.75 ± 0.02

**Table 12:** Estimated values of the Gini index (*g*) and Kolkata index (*k*) as measures of inequality in death counts resulting from natural disasters such as earthquakes and tsunamis (adapted from [29]). The data presented here provide valuable insights into the distribution of fatalities resulting from natural disasters and the effectiveness of these inequality indices in capturing the severity of such events.

Type of Disaster	*g* Index	*k* Index
Earthquake	0.94±0.02	0.95±0.02
Flood	0.98±0.02	0.98±0.02
Tsunami	0.93±0.02	0.94±0.02

**Table 13:** Inequality statistics of Olympic medals and winning countries (2008–2020). Typically, the number of medals (gold, silver, or bronze) for each year is about 300, with around 200 contesting countries. When the cumulative fraction of medals is plotted against the fraction of countries winning them (ordered from the fewest to most medals won), the result is the Lorenz curve, based on which we estimate the Gini (*g*) and Kolkata (*k*) indices for each year. As the level of competition is extremely high in the Olympics (with no welfare support for equality in achievements among contestants), typically, we find 13 to 16 percent of countries win 84–87 percent of medals (gold, silver, or bronze) in any Olympics.

Year	Medal	*g*	*k*
2020	Gold	0.87	0.85
	Silver	0.86	0.85
	Bronze	0.84	0.84
	Total	0.84	0.83
2016	Gold	0.88	0.87
	Silver	0.86	0.85
	Bronze	0.85	0.85
	Total	0.85	0.84
2012	Gold	0.89	0.87
	Silver	0.87	0.85
	Bronze	0.84	0.84
	Total	0.85	0.85
2008	Gold	0.89	0.87
	Silver	0.85	0.84
	Bronze	0.86	0.85
	Total	0.85	0.84

## Data Availability

The data will be available on request to the corresponding author.
